# Highly tunable TetR-dependent target gene expression in the acetic acid bacterium *Gluconobacter oxydans*

**DOI:** 10.1007/s00253-021-11473-x

**Published:** 2021-08-27

**Authors:** Philipp Moritz Fricke, Martha Lürkens, Max Hünnefeld, Christiane K. Sonntag, Michael Bott, Mehdi D. Davari, Tino Polen

**Affiliations:** 1 Forschungszentrum Jülich GmbH, IBG-1: Biotechnology, Institute of Bio- and Geosciences, 52425 Jülich, Germany; 2 RWTH Aachen University, Institute of Biotechnology, Worringerweg 3, 52074 Aachen, Germany; 3 Department of Bioorganic Chemistry, Leibniz Institute of Plant Biochemistry, Weinberg 3, 06120 Halle, Germany

**Keywords:** Promoter, Induction, Expression, Plasmid, mNeonGreen, Membrane-bound dehydrogenase

## Abstract

**Abstract:**

For the acetic acid bacterium (AAB) *Gluconobacter oxydans* only recently the first tight system for regulatable target gene expression became available based on the heterologous repressor-activator protein AraC from *Escherichia coli* and the target promoter P_*araBAD*_. In this study, we tested pure repressor-based TetR- and LacI-dependent target gene expression in *G. oxydans* by applying the same plasmid backbone and construction principles that we have used successfully for the *araC*-P_*araBAD*_ system. When using a pBBR1MCS-5-based plasmid, the non-induced basal expression of the Tn*10*-based TetR-dependent expression system was extremely low. This allowed calculated induction ratios of up to more than 3500-fold with the fluorescence reporter protein mNeonGreen (mNG). The induction was highly homogeneous and tunable by varying the anhydrotetracycline concentration from 10 to 200 ng/mL. The already strong reporter gene expression could be doubled by inserting the ribosome binding site AGGAGA into the 3’ region of the P_*tet*_ sequence upstream from *mNG*. Alternative plasmid constructs used as controls revealed a strong influence of transcription terminators and antibiotics resistance gene of the plasmid backbone on the resulting expression performance. In contrast to the TetR-P_*tet*_-system, pBBR1MCS-5-based LacI-dependent expression from P_*lacUV5*_ always exhibited some non-induced basal reporter expression and was therefore tunable only up to 40-fold induction by IPTG. The leakiness of P_*lacUV5*_ when not induced was independent of potential read-through from the *lacI* promoter. Protein-DNA binding simulations for pH 7, 6, 5, and 4 by computational modeling of LacI, TetR, and AraC with DNA suggested a decreased DNA binding of LacI when pH is below 6, the latter possibly causing the leakiness of LacI-dependent systems hitherto tested in AAB. In summary, the expression performance of the pBBR1MCS-5-based TetR-P_*tet*_ system makes this system highly suitable for applications in *G. oxydans* and possibly in other AAB.

**Key Points:**

• *A pBBR1MCS-5-based TetR-P*_*tet*_* system was tunable up to more than 3500-fold induction*.

• *A pBBR1MCS-5-based LacI-P*_*lacUV5*_* system was leaky and tunable only up to 40-fold*.

• *Modeling of protein-DNA binding suggested decreased DNA binding of LacI at pH < 6*.

**Supplementary Information:**

The online version contains supplementary material available at 10.1007/s00253-021-11473-x.

## Introduction

The acetic acid bacterium (AAB) *Gluconobacter oxydans* harbors the beneficial ability of regio- and stereoselective incomplete oxidation of a variety of substrates (e.g., sugars and sugar alcohols) in the periplasm by membrane-bound dehydrogenases (mDHs) and release of resulting products into the cultivation medium (Mamlouk and Gullo 2013; Mientus et al. 2017; Pappenberger and Hohmann 2014). Because of this feature, *G. oxydans* is industrially used for oxidative biotransformations of carbohydrates to produce e.g. the vitamin C precursor l-sorbose, the tanning lotion additive dihydroxyacetone, and 6-amino-l-sorbose used for the production of the antidiabetic drug miglitol (Ameyama et al. 1981; Gupta et al. 2001; Hekmat et al. 2003; Saito et al. 1997; Tkac et al. 2001; Wang et al. 2016).

For the expression of target genes in *G. oxydans*, so far only constitutive promoters have been used due to the lack of a regulatable promoter demonstrated to be functional and tunable in *G. oxydans* (reviewed in Fricke et al. 2021). Only recently the first tight system became available for tunable induction of gene expression in *G. oxydans*. This system is based on AraC-P_*araBAD*_ and the induction by l-arabinose binding to the regulator protein AraC (Fricke et al. 2020). AraC typically represses the target promoter P_*araBAD*_ by DNA bending in the absence of the inducer l-arabinose and activates it in the presence of the inducer by a modified binding to the promoter DNA and thereby releasing the bending (Schleif 2010). However, in *G. oxydans*, P_*araBAD*_ is almost not active in the absence of *araC* and thus, repression by AraC is not required in *G. oxydans* for tightness of P_*araBAD*_ in the absence of the inducer (Fricke et al. 2020). In contrast, in *Gluconacetobacter* and *Komagataeibacter* plasmid-based AraC-P_*araBAD*_ was reported to be very leaky (Teh et al. 2019). While AraC typically is acting both as repressor and activator, the transcriptional regulators TetR and LacI only exert a repressor function and dissociate from their operator DNA when forming a complex with their respective inducer (Hillen et al. 1983; Khoury et al. 1991; Miller 1970; Sellitti et al. 1987; Wray and Reznikoff 1983). P_*tet*_ and P_*lac*_ then enable transcription of the downstream gene by RNA polymerase. In AAB both TetR- and LacI-dependent target gene expression have so far been reported to be very leaky or inhomogeneously induced. In *Komagataeibacter rhaeticus* iGEM, the TetR-P_*tet*_ system from transposon Tn*10* exhibited only approximately 1.5-fold induction due to high leakiness in the absence of the inducer anhydrotetracycline (Florea et al. 2016). With a LacI-based expression system in *K. xylinus*, the induction ratio also appeared to be low and only a fraction of cells showed some induction (Liu et al. 2020). For another LacI-based system, expression was also found to be very leaky in *G. oxydans* (Condon et al. 1991).

In this study, we aimed to test TetR- and LacI-dependent target gene expression in *G. oxydans* by applying the same plasmid backbone and construction principles that we have used recently for the construction of the *araC*-P_*araBAD*_ system (Fricke et al. 2020). With pBBR1MCS-5-based plasmid constructs, we found always some leakiness of the LacI-P_*lacuv5*_ system, yet extremely tight and optimally tunable target gene expression with TetR-P_*tet*_ making the latter system highly suitable for applications in *G.* *oxydans* and possibly in other AAB.

## Materials and methods

### Bacterial strains, plasmids, and culture conditions

All strains and plasmids created and used in this study are listed in Table 1. *G. oxydans* was routinely cultivated in d-mannitol medium (pH 6) containing 4% (w/v) d-mannitol, 5 g L^−1^ yeast extract, 2.5 g L^−1^ MgSO_4_ × 7 H_2_O, 1 g L^−1^ (NH_4_)_2_SO_4_, 1 g L^−1^ KH_2_PO_4_ at 30 °C and 180 rpm, and supplemented with 50 µg mL^−1^ sodium cefoxitin. Besides d-mannitol and cefoxitin which were sterile filtered as stock solutions (20% (w/v) and 50 mg mL^−1^), all components were autoclaved for sterilization (120 °C, 20 min). Unless stated otherwise, for shake flask cultivations 10 mL or 60 mL of d-mannitol medium was inoculated from overnight precultures to an initial optical density at 600 nm (OD_600_) of 0.3 or 0.2 (UV-1800, Shimadzu) using 100 mL or 500 mL shaking flasks with three baffles. *G. oxydans* carrying pBBR1MCS-5- or pBBR1MCS-2-based plasmids were supplemented with 10 µg mL^−1^ gentamicin or 50 µg mL^−1^ kanamycin, respectively (Kovach et al. 1995). *Escherichia coli* strains were routinely grown at 37 °C and 160 rpm in lysogeny broth (LB) medium which was supplemented when appropriate with 10 µg mL^−1^ gentamicin or 50 µg mL^−1^ kanamycin. *G. oxydans* was transformed by conjugal transfer of plasmids from *E. coli* S17-1 (Kiefler et al. 2017). Competent *E. coli* cells were prepared by CaCl_2_ procedure and transformed as described (Hanahan 1983).

**Table 1:** Strains and plasmids used or constructed in this study

	Relevant characteristics	Reference / Source
Strain		
*E. coli* S17-1	Δ*recA*, *endA1*, *hsdR17*, *supE44*, *thi*-1, *tra*^+^	Simon et al. 1983
*G. oxydans* 621H	DSM 2343	DSMZ
Plasmid		
pBBR1MCS-5	Derivative of pBBR1MCS; Gm^R^	Kovach et al. 1995
pBBR1MCS-2	Derivative of pBBR1MCS; Km^R^	Kovach et al. 1995
pBBR1-tetall-strep_long	Derivative of pBBR1MCS-2 with *tetR*-P_*tet*_ fragment from *E. coli* Tn*10*	Gift from Uwe Deppenmeier, University of Bonn
pBBR1MCS-5-T_*gdhM*_-MCS-T_0028_	Derivative of pBBR1MCS-5 with terminator sequences of GOX0265 (T_*gdhM*_) and GOX0028 (T_0028_) flanking the multiple cloning site	This work
pBBR1MCS-5-T_*gdhM*_-*tetR*-P_*tet*_*-mNG*-T_BBa_B1002_-T_0028_	Derivative of pBBR1MCS-5-T_*gdhM*_-MCS-T_0028_ carrying fluorescent reporter gene *mNG* controlled by tetracycline-induced promoter P_*tet*_ and *tetR* encoding P_*tet*_ repressor TetR	This work
pBBR1MCS-5-*tetR*-P_*tet*_*-mNG*-T_BBa_B1002_-T_0028_	Derivative of pBBR1MCS-5-T_*gdhM*_-*tetR*-P_*tet*_*-mNG*-T_BBa_B1002_-T_0028_ lacking terminator T_*gdhM*_ downstream from *tetR*	This work
pBBR1MCS-5-T_*gdhM*_-*tetR*-P_*tet*_*-mNG*	Derivative of pBBR1MCS-5-T_*gdhM*_-*tetR*-P_*tet*_*-mNG*-T_BBa_B1002_-T_0028_ lacking terminators T_BBa_B1002_ and T_0028_ downstream from *mNG*	This work
pBBR1MCS-5-*tetR*-P_*tet*_*-mNG*	Derivative of pBBR1MCS-5-T_*gdhM*_-*tetR*-P_*tet*_*-mNG*-T_BBa_B1002_-T_0028_ lacking the terminators downstream from *tetR* and *mNG*	This work
pBBR1MCS-5-T_*gdhM*_-*tetR*-P_*tet*_*-*RBS-*mNG*-T_BBa_B1002_-T_0028_	Derivative of pBBR1MCS-5-T_*gdhM*_-*tetR*-P_*tet*_*-mNG*-T_BBa_B1002_-T_0028_ with ribosomal binding site AGGAGA (Hentschel et al. 2013) inserted upstream from *mNG*	This work
pBBR1MCS-5-T_*gdhM*_-P_*tet*_*-mNG*-T_BBa_B1002_-T_0028_	Derivative of pBBR1MCS-5-T_*gdhM*_-*tetR*-P_*tet*_*-mNG*-T_BBa_B1002_-T_0028_ lacking *tetR*	This work
pBBR1MCS-2-T_*gdhM*_-*tetR*-P_*tet*_*-mNG*-T_BBa_B1002_-T_0028_	Derivative of pBBR1MCS-2 carrying *mNG* controlled by P_*tet*_ and *tetR*, with each gene flanked by terminators	This work
pBBR1MCS-5-P_*lacI*_-*lacI*-P_*lacUV5*_*-*RBS-*mNG*-T_BBa_B1002_-T_0028_	Derivative of pBBR1MCS-5 carrying *mNG* with RBS AGGAGA controlled by IPTG-induced promoter P_*lacUV5*_ and *lacI* encoding P_*lacUV5*_ repressor LacI	This work
pBBR1MCS-5-P_*lacUV5*_*-*RBS*-mNG*-T_BBa_B1002_-T_0028_	Derivative of pBBR1MCS-5-P_*lacI*_-*lacI*-P_*lacUV5*_*-*RBS-*mNG-*tBBa-t28 lacking *lacI*	This work
pBBR1MCS-5-*lacI*-P_*lacI*_-P_*lacUV5*_*-*RBS-*mNG*-T_BBa_B1002_-T_0028_	Derivative of pBBR1MCS-5-P_*lacI*_-*lacI*-P_*lacUV5*_*-*RBS-*mNG*-T_BBa_B1002_-T_0028_ with opposite orientation of P_*lacI*_-*lacI* toward P_*lacUV5*_	This work

### Recombinant DNA work

All DNA oligonucleotides used for the construction of plasmids and sequencing are listed in Table S1 and were synthesized by Eurofins MWG. All restriction enzymes were purchased from Thermo Scientific. Polymerase chain reaction (PCR), restriction, and ligation reactions for DNA manipulations were conducted according to standard protocols (Sambrook et al. 1989). DNA fragments were amplified using Q5 polymerases according to the conditions recommended by the manufacturer (New England Biolabs). Unless stated otherwise for the construction of all reporter plasmids, amplified DNA fragments were integrated in the restricted broad-host vector pBBR1MCS-5 in a one-step isothermal Gibson assembly (50 °C, 1 h) (Gibson et al. 2009). All cloning steps to obtain desired plasmids were conducted in *E. coli* S17-1 and plasmids were isolated using a QIAprep spin miniprep kit (Qiagen). Inserts of all constructed plasmids were checked for correctness by DNA sequencing (Eurofins MWG).

### Construction of plasmids

For the construction of plasmids, the empty vector pBBR1MCS-5-T_*gdhM*_-MCS-T_0028_ was generated from pBBR1MCS-5. It carries the terminator sequences of GOX0265 (T_*gdhM*_) and GOX0028 (T_0028_) flanking the multiple cloning site (MCS) to minimize interfering effects between the plasmid backbone and expression of the inserted genes.

Plasmid pBBR1MCS-5-T_*gdhM*_-*tetR*-P_*tet*_*-mNG*-T_BBa_B1002_-T_0028_ was constructed using the primer pair PF1/PF2 to generate a 763 bp DNA fragment with *tetR*-P_*tet*_ from plasmid pBBR1-tetall-strep_long and primer pair PF3/PF4 to generate a 802 bp DNA fragment with *mNG* and the terminator BBa_B1002 from the iGEM parts library from pBBR1MCS-5-*araC*-P_*BAD*_*-mNG* (Fricke et al. 2020). For insertion of the two DNA fragments in pBBR1MCS-5-T_*gdhM*_-MCS-T_0028_, the plasmid was restricted with *Xba*I and *Eco*RI.

Plasmid pBBR1MCS-5-*tetR*-P_*tet*_*-mNG*-T_BBa_B1002_-T_0028_ lacking terminator T_*gdhM*_ downstream from *tetR* was constructed by amplification of a 576 bp DNA fragment from pBBR1MCS-5-T_*gdhM*_-*tetR*-P_*tet*_*-mNG*-T_BBa_B1002_-T_0028_ with primer pair PF5/PF6 and ligated with *Eco*81I/*Mun*I-digested pBBR1MCS-5-T_*gdhM*_-*tetR*-P_*tet*_*-mNG*-T_BBa_B1002_-T_0028_ replacing T_*gdhM*_-*tetR* by *tetR* only.

For the construction of pBBR1MCS-5-T_*gdhM*_-*tetR*-P_*tet*_*-mNG* lacking the terminators T_BBa_1002_ and T_0028_ downstream from *mNG*, the primer pairs PF7/PF8 and PF9/PF10 were used to amplify a 1,471 bp DNA fragment comprising *tetR*-P_*tet*_-*mNG* and a 643 bp DNA fragment comprising a part of the pBBR1MCS-5 backbone using pBBR1MCS-5-T_*gdhM*_-*tetR*-P_*tet*_*-mNG*-T_BBa_B1002_-T_0028_ as a template. Both DNA fragments were ligated with *Xba*I / *Bsp*1407I-digested pBBR1MCS-5-T_*gdhM*_-MCS-T_0028_.

The plasmid pBBR1MCS-5-*tetR*-P_*tet*_*-mNG* lacking all terminators downstream from *tetR* and *mNG* was generated by removing T_*gdhM*_ from pBBR1MCS-5-T_*gdhM*_-*tetR*-P_*tet*_*-mNG* with primer pair PF5/PF6 as described above for the removal of T_*gdhM*_ from pBBR1MCS-5-T_*gdhM*_-*tetR*-P_*tet*_*-mNG-*T_BBa_B1002_-T_0028*.*_

In another construct, for comparing and enhancing resulting reporter protein level when using the P_*tet*_ region including its native ribosome binding site (RBS_*Ptet*_) in *G.* *oxydans*, the RBS AGGAGA (RBS_AGGAGA_), functional and strong in *G.* *oxydans*, was inserted upstream from *mNG* (Fricke et al. 2020; Hentschel et al. 2013). Therefore, the DNA fragments *tetR*-P_*tet*_-RBS (761 bp) and RBS-*mNG* (811 bp) were amplified with the primer pairs PF1/PF12 and PF13/PF4, respectively, and ligated with *Xba*I/*Eco*RI-digested pBBR1MCS-5-T_*gdhM*_-MCS-T_0028_ to obtain plasmid pBBR1MCS-5-T_*gdhM*_-*tetR*-P_*tet*_-RBS-*mNG*-T_BBa_B1002_-T_0028_.

For plasmid pBBR1MCS-5-T_*gdhM*_-P_*tet*_*-mNG*-T_BBa_B1002_-T_0028_ lacking *tetR*, fragment P_*tet*_*-mNG* (901 bp) was amplified from pBBR1MCS-5-T_*gdhM*_-*tetR*-P_*tet*_*-mNG*-T_BBa_B1002_-T_0028_ with primer pair PF11/PF4 and ligated with *Xba*I / *Eco*RI-digested pBBR1MCS-5-T_*gdhM*_-MCS-T_0028_.

To change the plasmid backbone from pBBR1MCS-5 to pBBR1MCS-2 and create pBBR1MCS-2-T_*gdhM*_-*tetR*-P_*tet*_*-mNG*-T_BBa_B1002_-T_0028_, the DNA fragment with T_*gdhM*_-*tetR*-P_*tet*_*-mNG*-T_BBa_B1002_-T_0028_ was excised from pBBR1MCS-5-T_*gdhM*_-*tetR*-P_*tet*_*-mNG*-T_BBa_B1002_-T_0028_ and ligated with *Sac*I/*Xho*I-digested pBBR1MCS-2.

The plasmid pBBR1MCS-5-P_*lacI*_-*lacI*-P_*lacUV5*_-RBS-*mNG*-T_BBa_B1002_-T_0028_ was constructed by ligating the DNA fragment P_*lacI*_*-lacI*-P_*lacUV5*_*-lacZα-*RBS (1855 bp) and *mNG*-T_BBa_B1002_-T_0028_ (954 bp) with *Bsh*TI/*Sph*I-digested pBBR1MCS-5. Here, upstream from *mNG* the RBS AGGAGA known to be functional in *G.* *oxydans* was integrated into the construct (Hentschel et al. 2013). The DNA sequence of P_*lacI*_*-lacI*-P_*lacUV5*_*-lacZα* was derived from *E. coli* BL21 and obtained with primer pair PF14/PF15 and plasmid pK18*mobsacB*-DE3 (Kortmann et al. 2015). The DNA fragment *mNG*-T_BBa_B1002_-T_0028_ was amplified with primer pair PF16/PF17 from plasmid pBBR1MCS-5-T_*gdhM*_-*tetR*-P_*tet*_*-mNG*-T_BBa_B1002_-T_0028_.

For the construction of plasmid pBBR1MCS-5-P_*lacUV5*_-RBS-*mNG*-T_BBa_B1002_-T_0028_ lacking the repressor gene *lacI*, the DNA fragment P_*lacUV5*_-RBS-*mNG*-T_BBa_B1002_-T_0028_ (1575 bp) was amplified with primer pair PF18/PF17 from pBBR1MCS-5-P_*lacI*_-*lacI*-P_*lacUV5*_-RBS-*mNG*-T_BBa_B1002_-T_0028_ and ligated with *Bsh*TI/*Sph*I-digested pBBR1MCS-5.

The plasmid pBBR1MCS-5-*lacI*-P_*lacI*_-P_*lacUV5*_-RBS-*mNG*-T_BBa_B1002_-T_0028_ with P_*lacI*_-*lacI* in the opposite orientation to P_*lacUV5*_*-mNG* was constructed by amplification of the DNA fragments P_*lacI*_-*lacI* (1,261 bp) with the primer pair PF19/PF20 and P_*lacUV5*_*-mNG*-T_BBa_B1002_-T_0028_ (1556 bp) with the primer pair PF21/PF17. Both fragments were ligated into pBBR1MCS-5 digested with *Bsh*TI and *Sph*I.

### Measurements of mNG fluorescence

Expression of promoter–reporter constructs in *G. oxydans* was monitored using the fluorescence protein mNeonGreen (mNG) as a reporter (Shaner et al. 2013). In shake flask experiments, the inducibility of P_*tet*_-derived *mNG* expression in *G.* *oxydans* was tested by the addition of 200 ng mL^−1^ anhydrotetracycline (ATc) from a 0.2 mg mL^−1^ stock solution in 50% ethanol. Non-induced reference cultures were supplemented with an equal volume of 50% ethanol. For experiments with P_*lacUV5*_-controlled *mNG* expression, 1 mM of isopropyl-β-d-1-thiogalactopyranoside (IPTG) was supplemented from a 100 mM stock solution in water. An equal volume of water was added to non-induced reference cultures. Throughout the cultivation samples were taken to monitor growth (OD_600_) by a spectrophotometer (UV-1800, Shimadzu) and fluorescence emission using an Infinite M1000 PRO Tecan reader (λ_ex_ 504 nm / λ_em_ 517 nm, gain 60, ex/em bandwidth 5 nm, infinite M1000 PRO Tecan). Using a BioLector system, 800 µL batches of d-mannitol medium were inoculated from overnight starter cultures to an initial OD_600_ of 0.3 and incubated at 30 °C (1,200 rpm; 85% humidity) using 48-well Flowerplates® (m2p-labs). Backscattering light intensity (A_620 nm_) for growth and fluorescence emission (λ_ex_ 510 nm / λ_em_ 532 nm) were monitored online during the cultivation. Backscatter and fluorescence signals were measured using gain 15 or 20 and 50 or 60, respectively, as indicated in the figure legends. Fluorescence values that did not exceed the emission signals from cell-free control samples were set to 1. Specific fluorescence was calculated by taking the quotient of the fluorescence signal per biomass value at a given time point. Specific fluorescence values lower than in the cell-free control samples were set to 0.01. All data presented in the same graphs were obtained in the same BioLector growth experiment using identical gains.

### Cell flow cytometer analysis

Reporter gene expression was analyzed by measuring mNG fluorescence on the single cell level with *G.* *oxydans* 621H either carrying the plasmid pBBR1MCS-5-T_*gdhM*_-*tetR*-P_*tet*_*-mNG*-T_BBa_B1002_-T_0028_, pBBR1MCS-2-T_*gdhM*_-*tetR*-P_*tet*_*-mNG*-T_BBa_B1002_-T_0028_, or pBBR1MCS-5-P_*lacI*_*-lacI*-P_*lacUV5*_-RBS-*mNG*-T_BBa_B1002_-T_0028_ using a FACSAria™ Fusion cell sorter (BD Biosciences) run with 70 psi sheath pressure and equipped with a 70 µm nozzle. Data acquisition and analysis of the flow cytometer was controlled by the FACSDiva 8.0.3 software (BD Biosciences). Using a 488-nm solid blue laser beam, the forward scatter (FSC) and side scatter (SSC) were employed for cell analysis. Particles/events with FSC-H and SSC-H signals below a threshold of 200 a.u. and 300 a.u. were excluded from the analysis. Detection of emitted mNG fluorescence from the SSC signal was performed by combining a 502-nm long-pass and 530/30-nm band-pass filter. The entire cell population was analyzed in a three-step gating strategy. Initially, the assessed cell population was gated in a FSC-H *vs*. SSC-H plot, to exclude signals originating from electronic noise and cell debris. From the resulting population, the FSC-H signal was plotted against the FSC-W signal. Subsequently, the obtained population was gated in a SSC-H *vs*. SSC-W plot, to ensure singlet discrimination. The gated singlet population was used for fluorescence acquisition in all experiments (fluorescence intensity *vs.* cell count). For all samples, 100,000 events were recorded with an event rate below 10,000 events/s. FlowJo 10.7.2 for Windows (FlowJo, LLC) was used for data analysis and visualization of all gated events (*n* = 100,000).

### Fluorescence microscopy

For fluorescence microscopy, cells were placed on agarose-coated microscope slides and covered by a coverslip. Images were taken on a Zeiss AxioImager M2 imaging microscope that was equipped with a Plan-Apochromat 100 × /1.40-numerical aperture phase-contrast oil-immersion objective and an AxioCam MRm camera. Fluorescence was measured using the 46 HE filter set (λ_ex_ 500/20 nm / λ_em_ 535/30 nm). For all images, identical exposure times were applied. Digital images were acquired and analyzed with AxioVision Rel. 4.8 software (Zeiss).

### Total DNA extraction, library preparation, Illumina sequencing, and data analysis

Total DNA was purified from a culture aliquot using a NucleoSpin Microbial DNA Mini kit (MACHEREY–NAGEL). DNA concentrations were measured using a Qubit 2.0 fluorometer (Thermo Fisher Scientific). Illumina sequencing libraries of *tetR*-P_*tet*_ and *lacI*-P_*lacUV5*_ samples were prepared from 1 µg of isolated DNA using the NEBNext Ultra™ II DNA Library Prep Kit for Illumina according to the manufacturers’ instructions (NEB). The libraries were evaluated by qPCR using the KAPA library quantification kit (Peqlab) and then normalized for sample pooling. Paired-end sequencing with a read length of 2 × 150 bases was performed in-house on an Illumina MiSeq system. The demultiplexed sequencing output (base calls) was obtained as fastq files and used for trimming and quality filtering, mapping, and coverage calculation using the CLC Genomics Workbench software (Qiagen). For the mappings, the improved genome sequence from *G.* *oxydans* 621H and the *tetR*-P_*tet*_ or *lacI*-P_*lacUV5*_ plasmid sequence was used (Kranz et al. 2017).

### Computational methods

Homology modeling of TetR, LacI, and AraC structures was performed by YASARA Structure version 19.12.14 (Krieger et al. 2002) using the default settings (PSI-BLAST iterations: 6, E value cutoff: 0.5, templates: 5), and with oligomerization state adjusted to 2 for TetR and AraC, and to 4 for LacI (Altschul et al. 1997). A position-specific scoring matrix (PSSM) was used to score the obtained template structures (Jones 1999; Qiu and Elber 2006). The obtained hybrid models were further evaluated for protein geometry by VERIFY3D and ProSA (Eisenberg et al. 1997; Sippl 1993). Operator models of *tetO* and *araI*_*1*_ were generated with Avogadro as B-shaped DNA (Hanwell et al. 2012). The initial coordinates for *lacO* were taken from the X-ray structure (PDB ID 1EFA, resolution 2.6 Å) (Bell and Lewis 2000).

Surface residues were determined with PyMol script (default cutoff of 2.5 Å^2^). The protonation states of titratable residues were assigned on the basis of pK_a_ values obtained from the PROPKA 3.1 program for the pH values 7, 6, 5, and 4 (Olsson et al. 2011).

Modeling protein–protein and protein-DNA complexes was performed by using the HADDOCK Webserver (van Zundert et al. 2016). To understand the DNA binding behavior, protein-DNA docking simulations of the transcription factors and their respective operator sequence were done. In docking simulations, a dimeric model for TetR and LacI and a monomeric model for AraC were used. The charge states of proteins were adjusted to the respective pH based on the previous pK_a_ calculations and were adjusted to the respective pH either directly within the PDB file according to the HADDOCK specifications (Asp, Glu), or within the HADDOCK interface (His). Oligomerization of proteins was computed by performing protein–protein docking with the transcription factor subunits, i.e., monomer–monomer docking for TetR and AraC, and dimer-dimer docking for LacI. Due to the fact that the LacI homology model was missing the second tetramerization helix, the dimer structure taken from the crystal structure (PDB ID 3EDC, resolution 2.10 Å) was used for the LacI tetramerization modeling (Stenberg and Vihinen 2009).

## Results

### A pBBR1MCS-5-based TetR-P_*tet*_ system was very tight and highly inducible and tunable

The *tetR*-P_*tet*_ region including its native RBS from transposon Tn*10* was used to construct a pBBR1MCS-5-based reporter plasmid using the reporter gene *mNeonGreen* (*mNG*). The *tetR* gene under the control of its native promoter P_*tetR*_ overlapping with the divergently oriented TetR-dependent promoter P_*tet*_ followed by *mNG* were integrated into the MCS of pBBR1MCS-5-T_*gdhM*_-MCS-T_0028_ as described in Material and Methods. DNA sequences for transcription terminators were placed adjacent to the *tetR*-P_*tet*_*-mNG* insert to create transcriptional barriers between the genetic elements on the insert and on the plasmid backbone.

**Fig. 1:**
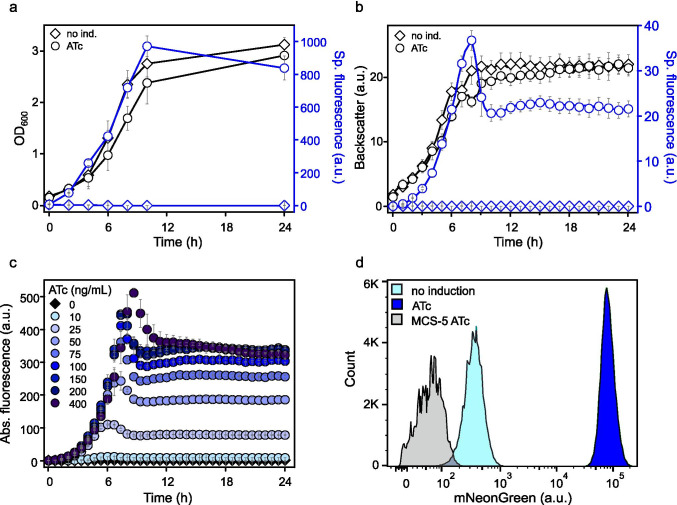
Performance of the TetR-P_*tet*_ system in *G. oxydans* 621H. **a** Growth (OD_600_) and specific mNG fluorescence in *G.* *oxydans* 621H carrying plasmid pBBR1MCS-5-T_*gdhM*_-*tetR*-P_*tet*_*-mNG*-T_BBa_B1002_-T_0028_ in ATc-induced (200 ng mL^−1^) and non-induced condition in shake flasks. The mNG fluorescence was measured in a Tecan reader (gain 60). The specific fluorescence was calculated from absolute fluorescence per OD_600_. Data represent mean values and standard deviation from three biological replicates with three technical replicates each. **b** Growth according to backscatter (gain 15) and specific mNG fluorescence (with gain 50) of *G.* *oxydans* 621H carrying plasmid pBBR1MCS-5-T_*gdhM*_-*tetR*-P_*tet*_*-mNG*-T_BBa_B1002_-T_0028_ in ATc-induced (200 ng mL^−1^) and non-induced condition in microscale BioLector cultivations. Data represent mean values and standard deviation from four biological replicates with three technical replicates each. **c** Graded ATc-dependent *mNG* expression in *G. oxydans* 621H carrying plasmid pBBR1MCS-5-T_*gdhM*_-*tetR*-P_*tet*_*-mNG*-T_BBa_B1002_-T_0028_ in microscale BioLector cultivations. Reporter gene expression measured as fluorescence (gain 50) was induced with increasing concentrations of ATc from 10 to 400 ng mL^−1^ as indicated. **d** FACS analysis of *G.* *oxydans* 621H carrying plasmid pBBR1MCS-5-T_*gdhM*_-*tetR*-P_*tet*_*-mNG*-T_BBa_B1002_-T_0028_ or empty vector pBBR1MCS-5 (MCS-5) as a control. Cells were grown in shake flasks with d-mannitol medium without and with 200 ng mL^−1^ ATc. FACS analysis was performed 7 h after inoculation/induction. Total counts per sample represent 100.000 events

The leakiness and inducibility of P_*tet*_ were tested in *G. oxydans* 621H harboring the plasmid pBBR1MCS-5-T_*gdhM*_-*tetR*-P_*tet*_*-mNG*-T_BBa_B1002_-T_0028_ by omitting and adding anhydrotetracycline (ATc). A pre-culture was split and used to inoculate shake flasks for growth in d-mannitol medium without and with 200 ng mL^−1^ ATc. The highest mNG fluorescence signals were measured in induced cultures after 10 h of growth at the end of the exponential growth phase, followed by a slight decrease in the stationary phase (Fig. 1a). In the non-induced cultures, the mNG signals barely surpassed background signals of cell-free control samples, suggesting very tight repression of P_*tet*_ in the absence of inducer. Based on the absolute and the specific mNG fluorescence, the maximal induction ratios were calculated to be 2284 ± 263-fold and 2661 ± 180-fold, respectively. At the end of the cultivation (24 h), cells of an induced culture were harvested and total DNA was purified for Illumina sequencing. In the read data analysis, 99.5% of the reads mapped to the updated reference sequences of the *G.* *oxydans* 621H genome, the 5 endogenous plasmids, and the mNG expression plasmid with *tetR*-P_*tet*_ (Kranz et al. 2017). Thus, the sequencing results excluded undesired contaminations and verified that the P_*tet*_-derived *mNG* expression was highly induced in *G.* *oxydans* 621H carrying the plasmid pBBR1MCS-5-T_*gdhM*_-*tetR*-P_*tet*_*-mNG*-T_BBa_B1002_-T_0028_. In microscale BioLector cultivations a similar induction profile of P_*tet*_ was observed as in shake flasks. The fluorescence in ATc-supplemented cultures peaked approximately after 8 h also followed by a decreased fluorescence level in the stationary phase (Fig. 1b). Again, the basal expression under non-induced conditions was barely detectable and the maximal induction ratio based on the specific fluorescence was calculated to be 3674 ± 193-fold. The drop in mNG fluorescence signals observed in shake flasks and in BioLector cultivations in the stationary phase was caused by the decreased pH of the medium (pH 4.6) and could be largely recovered at pH 6 as outlined below and described previously (Fricke et al. 2020).

To verify that TetR is responsible for the repression of P_*tet*_ also in *G. oxydans*, we constructed the plasmid pBBR1MCS-5-T_*gdhM*_-P_*tet*_*-mNG*-T_BBa_B1002_-T_0028_ lacking the *tetR* gene. In the absence of the plasmid-encoded regulator TetR, in both conditions with ATc supplement and without, the *mNG* expression from P_*tet*_ was even higher than in the induced control cultures containing the *tetR* gene on the plasmid (Fig. S1). This indicated that in *G. oxydans* P_*tet*_ is also fully repressed by TetR like in *E. coli* and that non-blocked P_*tet*_ is highly active in *G.* *oxydans*. Interestingly, even without the *tetR* gene the addition of ATc resulted in a higher maximal specific fluorescence (28.8 ± 1.3 a.u.) than without ATc (23.5 ± 1.9 a.u). This suggested that in *G.* *oxydans* the P_*tet*_ activity could additionally be affected by an endogenous factor.

We then tested the tunability of the TetR-P_*tet*_ system in *G.* *oxydans* by varying the ATc concentration from 10 to 400 ng mL^−1^. Gradual increase of the inducer concentration led to a gradual increase in mNG fluorescence illustrating the high dynamic range of P_*tet*_-derived gene expression in *G.* *oxydans* with the pBBR1MCS-5 backbone (Fig. 1c). Comparing the peaking fluorescence signals, the system appeared to be almost fully induced with 150 to 200 ng mL^−1^ ATc. According to flow cytometry analysis (FACS) of samples taken 7 h after induction (Fig. 1a), in both ATc-induced and non-supplemented cultures high population homogeneity was observed (Fig. 1d). Ninety-five percent and 94% of the cells of a sample were found to exhibit either very low (~ 400 a.u., non-induced) or very high (~ 80,000 a.u., ATc-induced) mNG fluorescence signals. To visualize this high homogeneity by microscopy, images of induced and non-induced *G.* *oxydans* cells harboring the plasmid pBBR1MCS-5-T_*gdhM*_-*tetR*-P_*tet*_*-mNG*-T_BBa_B1002_-T_0028_ were taken (Fig. S2). In accordance with the data obtained by flow cytometry, the microscopic images confirmed the strong inducibility of pBBR1MCS-5-based TetR-P_*tet*_ and the highly homogenous induction response in *G.* *oxydans*.

During growth, the *G. oxydans* d-mannitol medium initially set to pH 6 is acidified to pH 4.7 which causes a loss of intracellular mNG fluorescence suggesting a decreased cytoplasmic pH, at least in the stationary phase (Fricke et al. 2020). We wanted to test experimentally whether an already initially lower medium pH could result in leakiness of P_*tet*_ in *G.* *oxydans* already during growth as observed in *Komagataeibacter* (Florea et al. 2016). Therefore, *G.* *oxydans* carrying the plasmid pBBR1MCS-5-T_*gdhM*_-*tetR*-P_*tet*_*-mNG*-T_BBa_B1002_-T_0028_ was grown in d-mannitol medium initially adjusted to pH 6, 5, 4 and 3 both in the absence and presence of ATc. After 23 h cells were centrifuged and resuspended in fresh d-mannitol-free medium set to pH 6 to check for potential pH-dependent recovery of mNG fluorescence above the respective levels monitored before that would indicate a leakiness of P_*tet*_ together with a loss of mNG fluorescence during growth. In all non-induced cultures, very low maximal fluorescence signals (0.6 ± 0.1 to 1.3 ± 0.2 a.u.) were measured independent of the initial medium pH (Fig. S3a-b). Cells grown in pH 3 exhibited the lowest signals and a different growth according to backscatter compared to all other cultures. No sufficient increase of mNG fluorescence was observed in any non-induced condition in the exponential growth phase to suggest a leakiness of P_*tet*_ in *G.* *oxydans*. Moreover, after transfer (23 h) of the cells into fresh d-mannitol-free medium adjusted to pH 6 no recovery of mNG fluorescence above the levels before could be observed. Thus, since the strong induction of P_*tet*_ by ATc in the cells grown in each medium of this pH series indicated that the mNG protein could always be produced during the exponential phase of growth and exhibited strong fluorescence property (Fig. S3c-d), the results together indicated that expression from P_*tet*_ was really tightly repressed in *G.* *oxydans* in all pH conditions, even in medium initially set to pH 3.

### Terminators strongly affected mNG expression strength but not repression of P_*tet*_

**Fig. 2:**
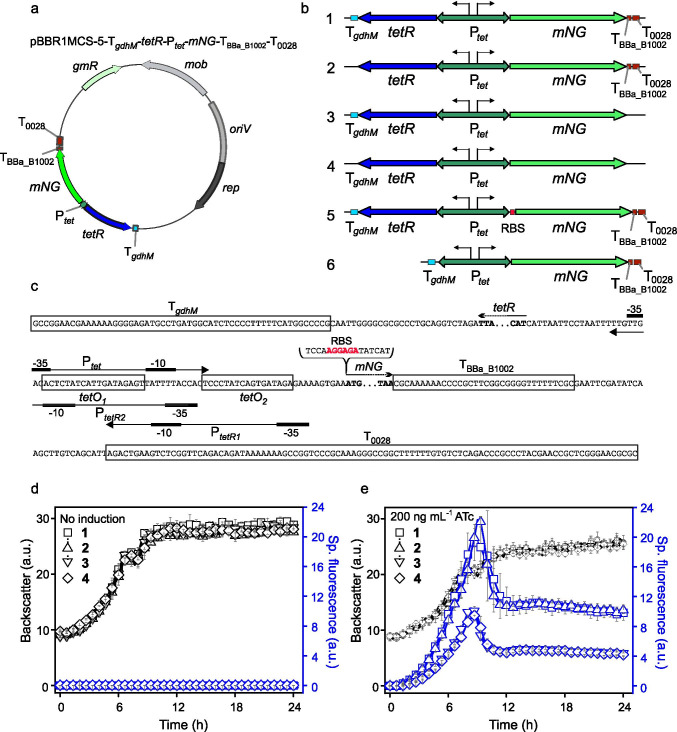
Variants of pBBR1MCS-5-based expression plasmids with *tetR*-*P*_*tet*_ and reporter gene expression in *G.* *oxydans* 621H. **a** Map of plasmid pBBR1MCS-5-T_*gdhM*_-*tetR*-P_*tet*_*-mNG*-T_BBa_B1002_-T_0028_ carrying the fluorescence reporter gene *mNeonGreen* (*mNG*) expressed from P_*tet*_ with the adjacent *tetR* gene and terminators T_*gdhM*_, T_BBa_B1002_ and T_0028_. **b** Variants of the plasmid insert with reporter gene *mNG* to test TetR-P_*tet*_-dependent expression in the presence and absence of terminators downstream from *tetR* and *mNG*, with RBS AGGAGA (Hentschel et al. 2013) inserted in the 3’ region of P_*tet*_ upstream from *mNG*, and without *tetR*. **c** DNA sequence details with P_*tet*_ region and TetR binding sites (*tetO1* and *tetO2*) and terminator sequences adjacent to *tetR* and *mNG*. **d + e**) Growth according to backscatter and specific mNG fluorescence in *G. oxydans* carrying either plasmid pBBR1MCS-5-T_*gdhM*_-*tetR*-P_*tet*_*-mNG*-T_BBa_B1002_-T_0028_ (1), or the plasmid lacking T_*gdhM*_ (2) or T_BBa_B1002_-T_0028_ (3) or all terminators (4) under non-induced (d) and ATc-induced (**e**) condition in microscale BioLector cultivations. For induction 200 ng mL^−1^ ATc was present in the d-mannitol medium. Data represent mean values and standard deviation from two biological replicates with three technical replicates each. T_*gdhM*_: terminator sequence of *gdhM* (GOX0265); T_0028_: terminator sequence of GOX0028. BioLector settings: backscatter gain 20, fluorescence gain 50

Since we did not observe and could not show leakiness of the TetR-P_*tet*_ system in growing *G.* *oxydans* cells *per se* or in dependence of the medium pH, we wanted to analyze the influence of terminator sequences on the functionality and leakiness of the TetR-P_*tet*_ system. Therefore, we constructed plasmids lacking terminators downstream from *tetR* or *mNG* and both and compared these constructs in regard to P_*tet*_-derived *mNG* expression in *G.* *oxydans* (Fig. 2). The terminators downstream from *mNG* had a major influence on the resulting mNG fluorescence level. With plasmid pBBR1MCS-5-T_*gdhM*_-*tetR*-P_*tet*_*-mNG* not having T_BBa_B1002_ and T_0028_ downstream from *mNG*, the mNG fluorescence signals were reduced by half in ATc-induced *G.* *oxydans* cells. Compared to the reference plasmid including all terminators, the maximum in specific fluorescence dropped from 19.4 ± 2.9 a.u. to 10.5 ± 0.2 a.u. (Fig. 2e). The specific fluorescence signals of *G.* *oxydans* harboring the plasmid pBBR1MCS-5-*tetR*-P_*tet*_*-mNG*-T_BBa_B1002_-T_0028_ lacking T_*gdhM*_ downstream from *tetR* were significantly (*p* = 0.0383) higher (22.3 ± 1.1 a.u.) compared to the reference (19.4 ± 2.9 a.u.) suggesting a little positive effect on P_*tet*_-derived induction and expression strength when T_*gdhM*_ downstream from *tetR* was absent. The construct pBBR1MCS-5-*tetR*-P_*tet*_*-mNG* lacking all terminators performed similar (9.5 ± 0.7 a.u.) as pBBR1MCS-5-T_*gdhM*_-*tetR*-P_*tet*_*-mNG* lacking only the terminators downstream from *mNG* (10.5 ± 0.2 a.u.)*.* Hence, in *G.* *oxydans* and in case of *mNG,* termination of the target gene transcription close to the 3’ end might be important to achieve higher expression levels in *G. oxydans.* None of the terminators appeared to affect the repression of P_*tet*_ in the absence of ATc and a P_*tet*_ leakiness was not observed.

### Comparison of the pBBR1MCS-2 / -5 backbones and insertion of AGGAGA as RBS

The broad-host-range vectors pBBR1MCS-5 and pBBR1MCS-2 differ in the antibiotics resistance they convey for clone selection. pBBR1MCS-5 *gmR* encodes for a gentamicin-3-acetyltransferase enabling growth on gentamicin and pBBR1MCS-2 *neoR*/*kanR* encodes for a neomycin/kanamycin phosphotransferase enabling growth on kanamycin (Kovach et al. 1995). Furthermore, *gmR* and *neoR*/*kanR* are located on different DNA strands which in the case of *neoR*/*kanR* could result in transcripts partially overlapping with and antisense to the mRNA of the target gene present in the cloned insert. To check whether the plasmid backbone and antibiotics resistance affect the inducibility of P_*tet*_ in *G.* *oxydans* we compared the performance of the TetR-P_*tet*_ system with pBBR1MCS-5 and pBBR1MCS-2.

**Fig. 3:**
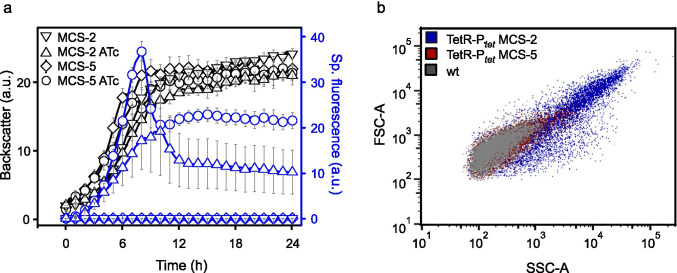
Comparison of pBBR1MCS-5 and pBBR1MCS-2 with *tetR*-P_*tet*_. **a** Growth according to backscatter (gain 15) and specific mNeonGreen (mNG) fluorescence (gain 50) in *G. oxydans* 621H carrying either plasmid pBBR1MCS-5-T_*gdhM*_-*tetR*-P_*tet*_*-mNG*-T_BBa_B1002_-T_0028_ or pBBR1MCS-2-T_*gdhM*_-*tetR*-P_*tet*_*-mNG*-T_BBa_B1002_-T_0028_ in microscale BioLector cultivations. For induction, always 200 ng mL^−1^ ATc was present in the d-mannitol medium. Data represent mean values and standard deviation from at least three biological replicates with three technical replicates each. **b** Cell morphology according to FACS analysis of *G.* *oxydans* 621H type strain (wt) without plasmid and with either the pBBR1MCS-5- or pBBR1MCS-2-based *tetR*-P_*tet*_ system, all without the inducer ATc

In *G. oxydans* the pBBR1MCS-2-based TetR-P_*tet*_ system performed much worse compared to the pBBR1MCS-5-based system despite the similarities of the two plasmids. With pBBR1MCS-2, the specific mNG fluorescence with induced P_*tet*_ was almost halved and reached 19.2 ± 9.7 a.u. ~ 10 h after induction at the end of the exponential phase of growth, while with pBBR1MCS-5 the maximal fluorescence peaked at 36.7 ± 2.2 a.u. after 8 h (Fig. 3a). Nevertheless, the maximal induction ratios were high with both pBBR1MCS-2 and pBBR1MCS-5 and were calculated to be 1915 ± 757 and 3674 ± 193, respectively. The basal expression under non-induced conditions was not affected by the plasmid backbone. Notably, a much higher standard deviation was obtained with pBBR1MCS-2. FACS analysis with gating of cells in regard to their volume and complexity by forward and side scattering (FSC and SSC) of light revealed that a major part of the *G.* *oxydans* cells with the pBBR1MCS-2 derivative and kanamycin exhibited a very different non-typical elongated cell morphology that was not observed with the pBBR1MCS-5 derivative and gentamicin (Fig. 3b). Approximately only 42% of the population with the pBBR1MCS-2 derivative passed the FACS gate where almost 100% of *G. oxydans* cells without plasmid or with the pBBR1MCS-5 derivative passed. This high heterogeneity of *G.* *oxydans* carrying the pBBR1MCS-2 derivative was not affected by the inducer ATc. The occurrence of a portion of elongated cells with pBBR1MCS-2 backbone and kanamycin was also demonstrated by fluorescence microscopy (Fig. S4). Several *G.* *oxydans* cells carrying pBBR1MCS-2 or pBBR1MCS-2-*tetR*-P_*tet*_*-mNG*-T_BBa_B1002_-T_0028_ appeared to be 10–15 µm and > 40 µm long, while the 621H reference cells typically were 2–3 µm. Apparently, the altered cell morphology had no direct effect on the inducibility of the TetR-P_*tet*_ system in *G.* *oxydans* as elongated cells also exhibited strong mNG fluorescence.

**Fig. 4:**
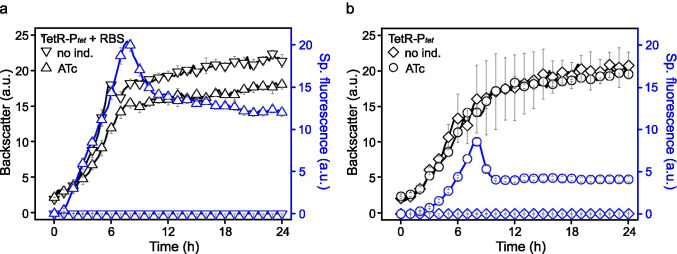
Influence of the ribosome binding site AGGAGA on mNG reporter expression with the TetR-P_*tet*_ system. Growth according to backscatter and specific mNeonGreen (mNG) fluorescence in *G. oxydans* 621H carrying plasmid pBBR1MCS-5-T_*gdhM*_-*tetR*-P_*tet*_-RBS-*mNG*-T_BBa_B1002_-T_0028_ with the RBS change (**a**) and with plasmid pBBR1MCS-5-T_*gdhM*_-*tetR*-P_*tet*_-*mNG*-T_BBa_B1002_-T_0028_ as the control (**b**) in microscale BioLector cultivations. Cells were grown in d-mannitol medium without or with 200 ng mL^−1^ ATc for induction. Data represent mean values and standard deviation from two biological replicates with three technical replicates each. BioLector settings: backscatter gain 20, fluorescence gain 40

To test improved translation and thus resulting reporter protein levels in *G.* *oxydans*, we also constructed a plasmid with the RBS AGGAGA that we had also used in the *araC*-P_*araBAD*_ system and inserted it upstream from *mNG* and downstream from *tetO*_*2*_ overlapping with the native RBS of the P_*tet*_ region (Fig. 2c). Thus, in this construct, RBS_*Ptet*_ and RBS_AGGAGA_ were consecutively upstream from *mNG*. The impact on reporter expression and inducibility was measured in BioLector cultivations. Grown in d-mannitol medium and induced with 200 ng mL^−1^ ATc, the fluorescence signals of induced cells were further increased and reached > 4000-fold induction, yet at such a high level that the detector gain in the BioLector had to be set from 50 down to 40 to avoid signal saturation. For both constructs with gain 40 setting, the insertion of RBS_AGGAGA_ doubled up the specific fluorescence in induced cells from 8.6 ± 0.4 a.u. for the reference to 20.4 ± 0.5 a.u. (Fig. 4). The basal expression in the absence of ATc was again extremely low, and the calculated maximal induction ratio based on the specific fluorescence with gain 40 was increased from 855 ± 7 for the reference to 2042 ± 1 for the plasmid with RBS_AGGAGA_.

### pBBR1MCS-5-based LacI-P_*lacUV5*_ was leaking and therefore tunable only up to 40-fold

**Fig. 5:**
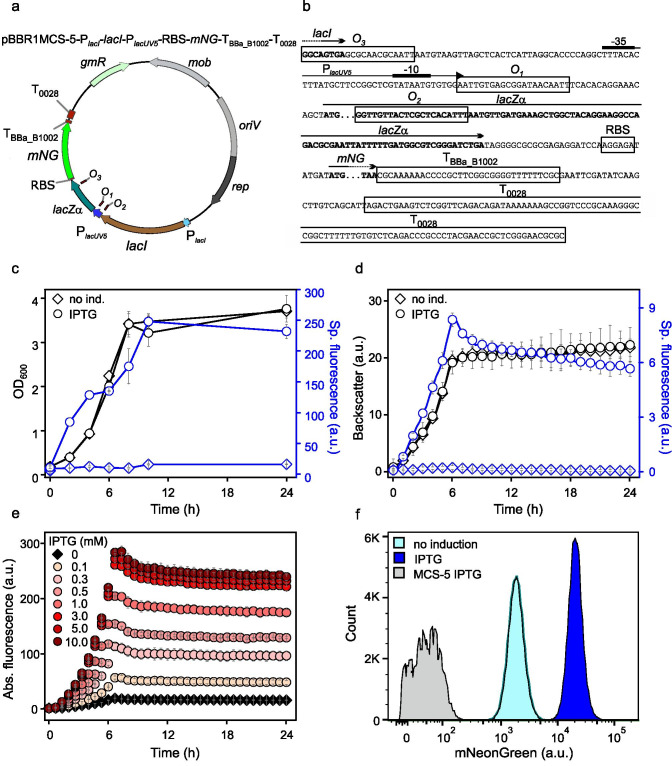
pBBR1MCS-5-based expression plasmid with *lacI*-*P*_*lacUV5*_ and reporter gene expression in *G.* *oxydans* 621H. **a** Map of the pBBR1MCS-5-based *lacI*-P_*lacUV5*_ plasmid. **b** Sequence information details of the *lacI*-P_*lacUV5*_ plasmid. The promoter P_*lacUV5*_ comprises the LacI operator sites *O*_*3*_, *O*_*1*_, and *O*_*2*_. The *O*_*3*_ sites overlaps with the 3’ end of *lacI*. The RBS AGGAGA was inserted upstream from *mNG*. The iGEM terminator sequence of BBa_B1002 was located downstream from *mNG* followed by terminator T_0028_ from GOX0028. **c** Shake flasks cultivations of *G.* *oxydans* 621H carrying plasmid pBBR1MCS-5-P_*lacI*_-*lacI*-P_*lacUV5*_-RBS-*mNG*-T_BBa_B1002_-T_0028_ in IPTG-induced (1 mM) and non-induced condition. The mNG fluorescence was measured in a Tecan reader (gain 60. The specific fluorescence was calculated from absolute fluorescence per OD_600_. **d** Microscale BioLector cultivations of *G.* *oxydans* 621H carrying plasmid pBBR1MCS-5-P_*lacI*_-*lacI*-P_*lacUV5*_-RBS-*mNG*-T_BBa_B1002_-T_0028_ in IPTG-induced (1 mM) and non-induced condition. All data represent mean values and standard deviation from three biological replicates with three technical replicates each. Backscatter gain 15, fluorescence gain 60. **e** Microscale BioLector cultivations of *G. oxydans* 621H carrying plasmid pBBR1MCS-5-P_*lacI*_-*lacI*-P_*lacUV5*_-RBS-*mNG*-T_BBa_B1002_-T_0028_ with increasing concentrations of IPTG as indicated. Fluorescence gain 60. **f** FACS analysis of *G.* *oxydans* 621H carrying plasmid pBBR1MCS-5-P_*lacI*_-*lacI*-P_*lacUV5*_-RBS-*mNG*-T_BBa_B1002_-T_0028_. Cells were grown in shake flasks with d-mannitol medium and 1 mM IPTG. FACS analysis was performed 7 h after inoculation and induction. Total counts per sample represent 100.000 events. As a control *G.* *oxydans* 621H carrying the empty vector pBBR1MCS-5 was used (MCS-5)

For the *lacI*-P_*lac*_ system, the insert containing *lacI* under control of its native promoter P_*lacI*_, promoter P_*lacUV5*_ and *lacZα* was derived from *E. coli* BL21(DE3). The promotor P_*lacUV5*_ differs from the native *E. coli* P_*lac*_ promoter by three point mutations leading to enhanced activity and reduced cyclic adenosine monophosphate (cAMP)-dependency for induction in *E. coli* (Hirschel et al. 1980). Downstream from the P_*lacUV5*_-controlled reporter gene *mNG*, the terminator T_BBa_B1002_ was placed and RBS_AGGAGA_ upstream from *mNG* (Fig. 5a, b)*.* The *lacI*-P_*lac*_ system includes three different operator sites which typically are bound by LacI in the absence of an inducer molecule to repress P_*lac*_-derived expression. Of these operator sites, *O*_*1*_ is located in close proximity to the -10 region of P_*lac*_, while *O*_*3*_ and *O*_*2*_ overlap with *lacI* and *lacZα*, respectively. In *E. coli* all three operator sites are bound by tetrameric LacI and are required for maximal repression (Oehler et al. 1990). Therefore, in order to enable full repression by LacI, the α-unit region of *lacZ* with *O*_*2*_ was included in the insert.

We examined the inducibility of P_*lacUV5*_ by IPTG in *G.* *oxydans* 621H harboring the plasmid pBBR1MCS-5-P_*lacI*_-*lacI*-P_*lacUV5*_-RBS-*mNG*-T_BBa_B1002_-T_0028_. Cells were grown in shake flasks using d-mannitol medium supplemented with and without 1 mM IPTG. After 10 h at the end of the exponential growth phase, the highest fluorescence signal was measured. Due to the basal expression in the absence of IPTG, the maximal induction ratios by the absolute and by the specific fluorescence were calculated to be 18 ± 4 and 18 ± 5, respectively (Fig. 5c). When testing the inducibility of P_*lacUV5*_ in microscale BioLector cultivations, a similar trend was observed. Under induced conditions, the fluorescence peaked approximately 6 h after induction and subsequently slightly decreased in the stationary phase while in non-induced cultures a basal fluorescence was measured (Fig. 5﻿d). Here, with 1 mM IPTG an average induction ratio of 41 ± 16 was calculated based on the specific fluorescence of the replicates in the BioLector. The P_*lacUV5*_-derived mNG expression exhibited a tunability by IPTG concentrations ranging from 0.1 to 10 mM in *G.* *oxydans* (Fig. 5e). The *mNG* expression from P_*lacUV5*_ was gradually increased up to 3 mM IPTG. Above 3 mM IPTG, the *mNG* expression hardly increased further and thus appeared to be saturated and best-calculated induction ratios were again in the range of 40-fold. Illumina sequencing data obtained with total DNA purified from induced cells showed that 99.5% of the reads mapped to the updated reference sequences of the *G.* *oxydans* 621H genome, the 5 endogenous plasmids, and the mNG expression plasmid with *lacI* (Kranz et al. 2017). Thus, the sequencing results verified that the P_*lacUV5*_-derived *mNG* expression was induced in *G.* *oxydans* carrying the intended plasmid pBBR1MCS-5-P_*lacI*_-*lacI*-P_*lacUV5*_-RBS-*mNG*-T_BBa_B1002_-T_0028_.

To analyze the induction of P_*lacUV5*_ in *G.* *oxydans* on the single cell level with regard to the homogeneity of *mNG* expression, flow cytometer analysis was applied (Fig. 5f). Within the chosen gate, 7 h after induction 95% of all induced cells exhibited high fluorescence (~ 11,000 a.u.) while under non-induced conditions in 96% of all cells, the fluorescence was at the somewhat leaky basal level (~ 1500 a.u.). This indicated that in the exponential growth phase, expression from induced P_*lacUV5*_ was very homogenous in *G.* *oxydans*. This was also supported by fluorescence microscopy (Fig. S5).

Similarly, as for TetR and P_*tet*_, we tested experimentally if initially lower medium pH values could affect the leakiness of P_*lacUV5*_. Therefore, *G.* *oxydans* carrying the plasmid pBBR1MCS-5-P_*lacI*_-*lacI*-P_*lacUV5*_-RBS-*mNG*-T_BBa_B1002_-T_0028_ was grown in d-mannitol medium initially adjusted to pH 6, 5, 4, and 3 in the absence and presence of IPTG. After 23 h, cells were centrifuged and resuspended in fresh d-mannitol-free medium of pH 6 to check for potential pH-dependent recovery of mNG fluorescence above the respective levels monitored before that would indicate a higher leakiness of P_*lacUV5*_ together with a previous loss of mNG fluorescence already during growth (Fig. S3e-h). In all non-induced cultures, overall basal fluorescence signals up to 10 a.u. were measured with decreasing maxima in the exponential phase when the initial medium pH decreased. Again, cells grown in pH 3 exhibited the lowest signals and a different growth according to backscatter compared to all other cultures. Compared to the pH 6 condition, no sufficient increase of mNG fluorescence was observed in any non-induced pH condition (6 h) in the exponential growth phase to suggest a higher leakiness of P_*lacUV5*_. Additionally, after the transfer (23 h) of the non-induced cells into fresh d-mannitol-free medium adjusted to pH 6 no recovery of mNG fluorescence above the levels with pH 6 could be observed in any condition. Interestingly, only for the non-induced pH 4 and pH 5 conditions, recovery of mNG fluorescence in pH 6 (24 h) above the respective maximal levels observed before in the exponential phase (6 h) were observed. This suggested that in the pH 4 and pH 5 conditions *G.* *oxydans* was not able to maintain its typical intracellular pH during growth and mNG fluorescence partially became inactive already in an early phase of growth (6 h).

### Strength of P_*lacUV5*_ was P_*lacI*_-lacI-dependent and leaking was independent of read-through

Unlike most operons, in the *E. coli lac* operon, the regulatory gene *lacI* is located immediately upstream from the target operon and is transcribed in the same direction. The repressor LacI forms tetramers binding in the absence of an inducer to up to three operator sites, thereby preventing transcription from the *lac* promoter directly downstream from *lacI*. Nevertheless, despite LacI binding and the presence of a *lacI* terminator region coinciding with the LacI operator site *O*_*1*_ directly downstream from the − 10 region, read-through is possible varying from 10 up to 80% (Horowitz and Platt 1982; Oehler et al. 1990). To check the possibility of read-through from P_*lacI*_ causing the leakiness observed in *G.* *oxydans* and interference by endogenous proteins on the P_*lacUV5*_ inducibility, we wanted to check and compare the pBBR1MCS-5-based reporter expression in the absence of P_*lacI*_-*lacI*, as well as with P_*lacI*_-*lacI* in opposite orientation toward P_*lacUV5*_.

Firstly, we constructed the plasmid pBBR1MCS-5-P_*lacUV5*_-RBS-*mNG*-T_BBa_B1002_-T_0028_ lacking P_*lacI*_ and most of the *lacI* gene, while keeping a short 3’ region of *lacI* to maintain all three operator sites of P_*lacUV5*_ including *O*_*3*_ partly overlapping with the end of the *lacI* gene. The analysis of the IPTG-dependent inducibility of P_*lacUV5*_ in *G.* *oxydans* in the absence of P_*lacI*_-*lacI* revealed no difference in fluorescence signals supplemented with IPTG or not (Fig. S6). This indicated that the inducibility observed in *G.* *oxydans* solely depended on derepression by LacI. Interestingly, compared to the original system with P_*lacI*_*-lacI*, the maximal reporter expression from P_*lacUV5*_ was strongly reduced from 8.4 ± 0.4 to 2.3 ± 0.2 according to the specific fluorescence (6–7 h) when P_*lacI*_*-lacI* was lacking. This suggested that a significant read-through from P_*lacI*_ appeared to contribute to the expression of the gene downstream from P_*lacUV5*_, thus increasing the apparent P_*lacUV5*_ strength in *G.* *oxydans* almost fourfold when induced.

Secondly, we constructed plasmid pBBR1MCS-5-*lacI*-P_*lacI*_-P_*lacUV5*_-RBS-*mNG*-T_BBa_B1002_-T_0028_ with P_*lacI*_-*lacI* in opposite direction to exclude potential read-through toward P_*lacUV5*_-mNG. Despite the opposite directions of P_*lacI*_ and P_*lacUV5*_, an even somewhat higher leakiness of P_*lacUV5*_ was observed when not induced (Fig. S7). Therefore, with 1 mM IPTG the maximal induction fold-change (6.4 h) calculated was only 15.4 ± 0.9 based on the specific fluorescence, while the original P_*lacI*_-*lacI*-P_*lacUV5*_ construct reached a maximal induction fold-change (6.7 h) of 39.7 ± 13.9. Surprisingly, the apparent induced P_*lacUV5*_ strength was restored according to the reporter fluorescence and reached the same levels as when P_*lacI*_-*lacI* were in the same direction as P_*lacUV5*_. Together, the results indicated that in *G.* *oxydans* the promoter strength of plasmid-based P_*lacUV5*_ appeared to depend on sequences further upstream and that the P_*lacUV5*_ leakiness was independent of potential read-through from P_*lacI*_.

### Modeling LacI and TetR binding to DNA at acidic pH values

Our results suggested that the leakiness of P_*lacUV5*_ in *G.* *oxydans* was independent of potential read-through from P_*lacI*_ and thus should result from transcription initiation from P_*lacUV5*_ and insufficient repression by LacI. Since the cytoplasmic pH of *G.* *oxydans* may readily be acidified to some extent already during growth when medium pH decreases, we modeled LacI- and TetR-DNA structures at different pH values using YASARA to predict if and how a potentially lower cytoplasmic hydrogen ion activity might affect the binding of LacI and TetR to DNA (Krieger et al. 2002). As a control, the AraC structure was also modeled since an l-arabinose-inducible AraC-P_*araBAD*_ system was shown to be fully functional in *G.* *oxydans* with plasmid pBBR1MSC-5, yet was very leaky in *Gluconacetobacter* and *Komagataeibacter* using another expression plasmid (Fricke et al. 2020; Teh et al. 2019). Protein-DNA bindings were simulated in two different steps for pH 7, 6, 5, and 4 (see supplementary Text S1 and S2).

**Fig. 6:**
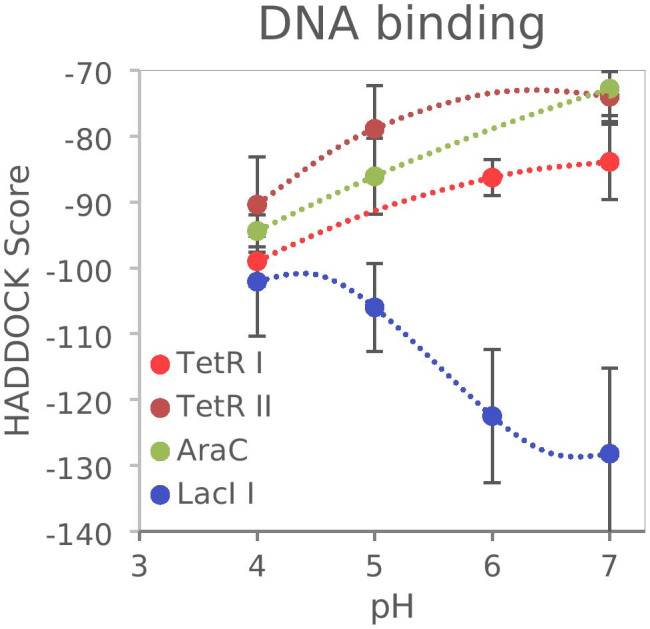
Prediction of DNA-binding behavior of LacI, TetR, and AraC at different pH values computed by the HADDOCK web server. Each data point corresponds to a cluster of docking poses. The lower (more negative) the HADDOCK score the energetically more favorable the docking pose. Trend lines plotted are based on clusters containing similar docking poses. For details see supplementary Text S1 and Text S2

Analysis of the HADDOCK simulations in different pH conditions showed that the HADDOCK scores of all five best-scoring clusters obtained in the TetR-DNA docking simulation for pH 7, 6, 5, and 4 lie within the standard deviation of each other. To make a prediction of the DNA binding behavior in dependence of the pH value, similar docking positions were identified for each pH value and the cluster scores were compared. It can be observed that with decreasing pH values the scores decrease. The smaller the score, i.e., the more negative, the better is the predicted binding to DNA, suggesting an increased binding of TetR to DNA (Fig. 6). Similar results were obtained for AraC. Thus, when comparing the scores of clusters with corresponding docking positions, the values generally decreased with decreasing pH values, suggesting a generally increased binding to DNA for both TetR and AraC (Fig. 6). On the contrary, for LacI unique clusters with correlating docking poses identified for pH 7, 6, 5, and 4 exhibited increasing HADDOCK scores for decreasing pH values, suggesting a decreased DNA binding of LacI when pH decreases (Fig. 6 and Supplementary Text S1 and S2).

## Discussion

In this study, we found with a pBBR1MCS-5-based TetR-P_*tet*_ system that inducible target gene expression based solely on de-repression of the heterologous target promoter can also perform extremely well in *G.* *oxydans*. With the pBBR1MCS-5-based plasmid constructed here, the anhydrotetracycline (ATc)-inducible promoter P_*tet*_ derived from the *E. coli* transposon Tn*10* exhibited excellently tunable expression performance in an inducer concentration-dependent manner with maximal induction ratios up to more than 3500-fold. This was due to extremely low basal reporter expression in the absence of an inducer, thus well repressed P_*tet*_, and P_*tet*_ being very strong in *G.* *oxydans*.

In contrast, expression from P_*lacUV5*_ was always leaky when not induced and this appeared to be independent of potential read-through from P_*lacI*_ which was located with *lacI* immediately upstream from P_*lacUV5*_ and, unlike most regulator genes and target operons, coupled very close and transcribed in the same direction. Therefore, the transcription termination by the native *lacI* terminator is expected to be very effective in *G.* *oxydans* and the P_*lacUV5*_ leakiness is rather related to the early events after transcription initiation from P_*lacUV5*_. In *E. coli*, the LacI repressor is not preventing *per se* the binding of RNA polymerase (RNAP), but is blocking the progress of RNAP which has bound to the DNA and has begun transcribing (Reznikoff et al. 1969). Transcription through roadblocks has been shown to depend on the cooperation of the leading and trailing RNAP molecules and thus depends on how many RNAP molecules are allowed to initiate from the same promoter upstream from the roadblock (Epshtein et al. 2003; Hao et al. 2014). In the case of P_*lac*_ or P_*lacUV5*_, the distance from the transcriptional start (TSS) to the LacI binding site *O*_2_ is rather short and site *O*_1_ is even overlapping with the TSS; thus, only one RNAP molecule is expected to initiate upstream from the roadblock. Additionally, the *E. coli* σ^70^ subunit of RNAP mediates a pause at the *lac* promoter closely downstream from the transcriptional start which is proposed to function in limiting the downstream gene expression (Nickels et al. 2004). If the *G.* *oxydans* σ^70^ subunit of RNAP also mediates this pause at the *lac* promoter is unknown. Nevertheless, RNAP cooperation should not be possible and thus not contribute to the leakiness of P_*lacUV5*_ in *G.* *oxydans*, yet the extent of read-through can also be affected by accessory factors. For example, in *E. coli* GreA and GreB rescue backtracked (roadblocked) RNAP by cleavage of the RNA, regenerating a new 3’ end at the catalytic site, and can aid passage of RNAP through a LacI roadblock in cells (Toulme et al. 2000). Furthermore, in *E. coli* the transcription-coupled repair protein Mfd binds to the DNA behind RNAP and uses ATP to push backtracked RNAP forward until the 3’ end of RNA is back at the catalytic center, yet the forces generated by Mfd may also result in RNAP termination (Park et al. 2002). *E. coli* GreA (158 aa, b3181) and *G.* *oxydans* GreA (157 aa, GOX0324) share 54% sequence identity, *E. coli* GreB (158 aa, b3406) and *G.* *oxydans* GreB (168 aa, GOX1860) share 47%, and *E. coli* Mfd (1148 aa, b1114) and *G.* *oxydans* Mfd (1173 aa, GOX0055) share 39%. It is unknown how these factors support roadblocked RNAP and read-through behind roadblocks in *G.* *oxydans* and AAB in general, yet the leakiness of P_*lac*_ or P_*lacUV5*_ is likely rather related to weaker binding of LacI to operator DNA and thus weaker promoter repression in *G.* *oxydans*.

Our docking simulations predicted that below pH 6 the binding of LacI to DNA is decreased which could cause or contribute to promoter leakiness. Neutralophilic bacteria maintain an intracellular pH between 7 and 8, while in acidophilic bacteria intracellular pH is considered to be maintained between 6 and 7 (Baker-Austin and Dopson 2007; Krulwich et al. 2011). The mannitol LB medium typically used for *G. oxydans* is initially set to pH 6 and can be acidified during growth in dependence of the carbon and energy sources, for example, to approximately pH 4.5 or 3.3 as well (Fricke et al. 2020). In such acidic pH conditions of the growth medium strongly decreased, intracellular fluorescence reporter activities of mNeonGreen (mNG) were observed in *G. oxydans* in the stationary phase (Fricke et al. 2020). Studies with isolated mNG revealed a strong decrease in fluorescence at pH 4.4 and below (Steiert et al. 2018). Therefore, the decrease in mNG fluorescence in *G.* *oxydans* cells was attributed to an acidified intracellular pH, at least in the stationary phase. The loss in mNG fluorescence in stationary *G. oxydans* cells can quickly be restored without protein synthesis just by short incubation in pH 6 condition as shown in this study and before (Fricke et al. 2020). Moreover, for the AAB *Acetobacter aceti*, the intracellular pH was reported to change from 5.8 to 3.9 already during growth when the medium pH acidified from 6.2 to 3.5 (Menzel and Gottschalk 1985). Despite intracellular pH maintenance mechanisms, it is conceivable that depending on the growth conditions in some or many AAB the cytoplasmic pH can decrease already during growth below the range of pH 6 to 7 considered to be typically maintained in acidophilic bacteria. According to our data, the mNG reporter fluorescence in *G.* *oxydans* cells with LacI-P_*lacUV5*_ grown in non-induced pH 4 and pH 5 conditions suggested that *G. oxydans* was not able to maintain its typical intracellular pH during growth and the mNG fluorescence became partially inactive already in the middle phase of growth (6 h). To get LacI-dependent systems tight and suitable for use in *G.* *oxydans* or AAB in general, the LacI protein may require engineering for increased binding to DNA in acidic pH condition. However, more intracellular pH data of AAB strains in various growth conditions are required. Besides leakiness, in *G.* *oxydans*, the strength of fully induced P_*lacUV5*_ was much lower compared to that of the fully induced P_*tet*_ (compare absolute fluorescence Fig. S3d and S3h). It was already reported before that P_*lac*_ is rather weak in *G.* *oxydans* 621H and the P_*lacUV5*_ mutant promoter exhibiting three base pair changes including two changes at positions − 9 and − 8 apparently also exhibits a similar low expression strength in *G.* *oxydans* (Hirschel et al. 1980; Kallnik et al. 2010). Thus, in *G.* *oxydans* the TetR-P_*tet*_ system will probably always be the choice for applications because of its extremely low basal expression and very high dynamic range of the tunable expression strength from P_*tet*_.

Interestingly, in *K.* *rhaeticus* iGEM, the TetR-P_*tet*_ system tested exhibited high leakiness in the absence of the inducer (Florea et al. 2016). In *G.* *oxydans*, we could neither observe nor demonstrate relevant leakiness of TetR-P_*tet*_, not with the pBBR1MCS-5-based construct variants (± terminators), not with the pBBR1MCS-2 backbone and kanamycin causing abnormal cell morphology, and not in the media of the pH series. Without terminators downstream from *mNG*, the length of the *mNG* transcript is assumed to exhibit an extended 3’ UTR. This appeared to only decrease transcript stability or translation, since the mNG signals were reduced to half, yet repression of P_*tet*_ was not affected. For the *tetR* transcript, it is unknown how stability and translation are affected in *G.* *oxydans* when the 3’ UTR is longer or shorter, yet altered repression of P_*tet*_ was also not observed with either of the constructs. A major difference of the two *tetR*-P_*tet*_ constructs tested in *K.* *rhaeticus* iGEM and in *G.* *oxydans* is that we maintained the opposite orientation of *tetR*-P_*tetR*_ and P_*tet*_ as originally present on Tn*10*, while in *K.* *rhaeticus* iGEM the construct tested contained the promoter J23118 or P_*lacI*_ for expression of *tetR* followed by a terminator sequence and all directly upstream from the target promoter P_*tet*_ and in the same direction as P_*tet*_ (Florea et al. 2016). It was assumed that the terminator is effective, yet it seemed to be unknown to what extend the terminator allowed potential read-through in these constructs. This could possibly cause the high leakiness of P_*tet*_ in *K.* *rhaeticus* iGEM, especially when cooperative RNAP molecules read through the TetR roadblock. On the other hand, our computational simulation also predicted that with decreasing pH value TetR dimerization decreases (Text S1 and Text S2). That could finally affect binding to DNA and thus promoter repression since TetR binds as a dimer (Hillen and Berens 1994). Thus, if the prediction holds true, in *G.* *oxydans* TetR dimerization is not sufficiently negatively affected by mild acidic intracellular pH. *Komagataeibacter* is typically grown in Hestrin − Schramm (HS) medium with glucose or in LGI medium with sucrose, both initially set to pH 6 or pH 4.5 and acidified further during growth (Florea et al. 2016; Hestrin and Schramm 1954). The intracellular pH of *A.* *aceti* was reported to change from 5.8 to 3.9 already during growth when the medium pH acidified from 6.2 to 3.5 (Menzel and Gottschalk 1985). The extent of being able to maintain the intracellular pH not only in the stationary phase but also during exponential growth could differ between AAB species. This could possibly be seen in the results with the LuxR-P_*lux*_ system in *Komagataeibacter* (Florea et al. 2016). The system exhibited condition-dependent induction only up to fivefold due to high leakiness, yet it also exhibited extremely low leakiness and much better induction performance according to fluorescence microscopic analysis in cells inside cellulose pellicles. Cellulose pellicles generate a micro-environment with the advantage of potentially protecting the cells from harsh conditions which could also include protection from acidic environmental pH. Again, to better understand the pH-dependent binding of regulators to DNA and to improve the expression performance of heterologous regulatable expressions systems so far leaky in AAB and their growth conditions, more intracellular pH data of AAB strains are required for various growth conditions. Additionally, experimental pH-dependent protein–protein and protein-DNA interaction data are needed.

In summary, we could show that in *G.* *oxydans* the TetR-P_*tet*_ system tested was extremely well performing with the pBBR1MCS-5 backbone and exhibited very strong reporter expression when induced, while the LacI-P_*lacUV5*_ system was always leaking and thus resulting in much lower induction ratios. Therefore, the pBBR1MCS-5-based TetR-P_*tet*_ system with the low inducer concentrations required for gradually tuning target gene expression appeared to be highly suitable for applications in *G.* *oxydans* and possibly in other AAB.

## Supplementary Information

Supplementary file1 (PDF 4.83 KB)

## Data Availability

Data and material are available upon request.
