# Supplementary material for: Highly tunable TetR-dependent target gene expression in the acetic acid bacterium Gluconobacter oxydans
Source: Appl Microbiol Biotechnol. 2021 Aug 27;105(18):6835–52. doi: 10.1007/s00253-021-11473-x (PMC8426231; doi:10.1007/s00253-021-11473-x)
Supplement: Supplementary file 1 — Supplementary file1 (PDF 4.83 KB) [file 253_2021_11473_MOESM1_ESM.pdf]

Journal: Applied Microbiology and Biotechnology

Highly tunable TetR-dependent target gene expression in the acetic acid bacterium *Gluconobacter oxydans*

Philipp Moritz Fricke[1], Martha Lürkens[2], Max Hünnefeld[1], Christiane K. Sonntag[1], Michael Bott[1], Mehdi D. Davari[3], and Tino Polen[1,*]

[1)] Forschungszentrum Jülich GmbH, IBG-1: Biotechnology, Institute of Bio- and Geosciences 52425 Jülich, Germany

[2)] RWTH Aachen University, Institute of Biotechnology, Worringerweg 3, 52074 Aachen, Germany

[3)] Department of Bioorganic Chemistry, Leibniz Institute of Plant Biochemistry, Weinberg 3, 06120 Halle, Germany

*** for correspondence:** Dr. Tino Polen t.polen@fz-juelich.de
Phone +49 2461 61 6205

Supplementary Data

Table S1 DNA oligonucleotides used in this study

Fig. S1 Expression from P_{tet} in the absence of the *tetR* gene
Fig. S2 Fluorescence microscopy of *G. oxydans* cells with TetR-P_{tet} system
Fig. S3 Influence of the initial medium pH on the repression of P_{tet} and P_{lacUV5}
Fig. S4 Fluorescence microscopy of *G. oxydans* cells with pBBR1MCS-2 derivative
Fig. S5 Fluorescence microscopy of *G. oxydans* cells with LacI-P_{lacUV5} system
Fig. S6 Expression from P_{lacUV5} in the absence of the *lacI* gene
Fig. S7 Expression from P_{lacUV5} with opposite orientation of P_{lacI}-*lacI*

Text S1 Generation and evaluation of TetR, LacI, and AraC homology models with Fig. S8-S10
Text S1.1 Evaluation of the TetR model with Fig. S11-S12
Text S1.2 Evaluation of the AraC hybrid homology model with Fig. S13-S14
Text S1.3 Evaluation of the LacI hybrid homology model with Fig. S15-S16
Text S2 Protein-DNA and protein-protein docking simulations with HADDOCK with Table S2
Text S2.1 DNA binding and dimerization behaviour of TetR for different pH values with Fig. S17-S26
Text S2.2 DNA binding and dimerization behaviour of AraC for different pH values with Fig. S27-S33
Text S2.3 DNA binding and oligomerization behaviour of LacI for different pH values with Fig. S34-S44

Table S1 DNA oligonucleotides used in this study

Name	DNA sequence 5’ > 3’
PF1	TTCATGGCCCCGCAATTGGGGCGCGCCCTGCAGGTCTAGATTAAGACCCACTTTCACATTTAAGTTGTTTTTCTAAT
PF2	TTCACCTTTAGACACCATTTCACTTTTCTCTATCACTGATAGGGAGT
PF3	TGATAGAGAAAAGTGAAATGGTGTCTAAAGGTGAAGAGGAC
PF4	GAGACTTCAGTCTAATGCTGACAAGCTTGATATCGAATTCGCGAAAAAACCCCGCCGAAG
PF5	CTCCACGGGGAGAGCCTGAGCAAACTGGCCTCAGGCATTTGAGAAGC
PF6	CTAGACCTGCAGGGCGCGCCCCAATTGGAGCTCCAATTCGCCCTATAGTG
PF7	TTCATGGCCCCGCAATTGGGGCGCGCCCTGCAGGTCTAGATTAAGACCCACTTTCACATTTAAGTTGTTTTTCTAAT
PF8	GGGCCCCCCCTCGAGTTACTTATACAGCTCATCCATGCCCATG
PF9	GAGCTGTATAAGTAACTCGAGGGGGGGCCC
PF10	CTTGCTGCTTGGATGCCCGAGGCATAGACTGTAC
PF11	GGCCCCGCAATTGGGGCGCGCCCTGCAGGTCTAGACATTAATTCCTAATTTTTGTTGACACTCTATC
PF12	ATGATATCTCCTTGGATTCACTTTTCTCTATCACTGATAGGGAGT
PF13	GAAAAGTGAATCCAAGGAGATATCATATGGTGTCTAAAGGTGAAGAGGAC
PF14	ACGGTCACACTGCTTCCGGTAGTCAATAAACCGGTGGCGCGCGAGAAC
PF15	CATATCATATCTCCTTGGATCCTCTCGCGCG
PF16	GCGCGAGAGGATCCAAGGAGATATGATATGGTGTCTAAAGG
PF17	CTTAATGAATTACAACAGTTTTTATGCATGCCTGGGTACCGGGCCC
PF18	CGGTCACACTGCTTCCGGTAGTCAATAAACCGGTGGCAGTGAGCGCAACG
PF19	ACGGTCACACTGCTTCCGGTAGTCAATAAACCGGTTCACTGCCCGCTTTCCAGTC
PF20	GTTGCGCTCACTGCCGGCGCGCGAGAACTGC
PF21	CAGTTCTCGCGCGCCGGCAGTGAGCGCAACGC

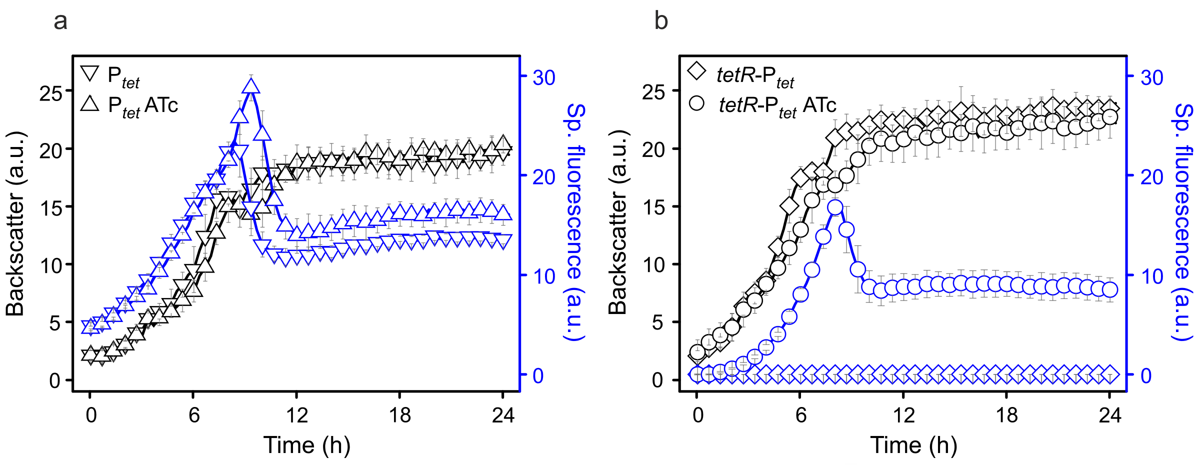

Fig. S1 Expression from P_{tet} in the absence of the *tetR* gene.

Growth according to backscatter and specific mNG fluorescence in *G. oxydans* 621H carrying plasmid pBBR1MCS-5-T_{gdhM}-P_{tet}-*mNG*-T_{BBa_B1002}-T_{0028} (**a**) or pBBR1MCS-5-T_{gdhM}-*tetR*-P_{tet}-*mNG*-T_{BBa_B1002}-T_{0028} (**b**) in ATc-supplemented and non-supplemented condition in microscale BioLector cultivations. The specific fluorescence was calculated from absolute fluorescence per backscatter. For induction, the D-mannitol medium contained 200 ng mL^{-1} ATc. Data represent mean values and standard deviation from three biological replicates with three technical replicates each. Backscatter gain 15; fluorescence gain 50.

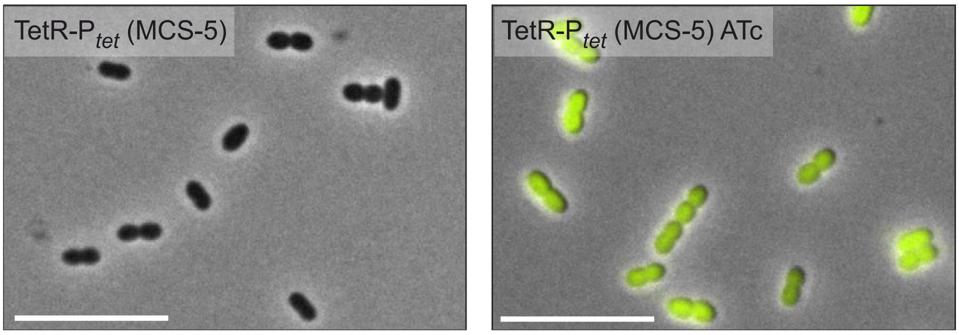

Fig. S2 Fluorescence microscopy of *G. oxydans* cells with TetR-P_{tet} system.

Representative merged images (bright field + fluorescence channel) of *G. oxydans* 621H cells harboring plasmid pBBR1MCS-5-T_{gdhM}-*tetR*-P_{tet}-*mNG*-T_{BBa_B1002}-T_{0028} in non-induced (left) and ATc-induced (right) condition 7 h after inoculation. Cells were grown in shake flask in D-mannitol medium without or with ATc (200 ng mL^{-1}). Size bars represent 10 µm.

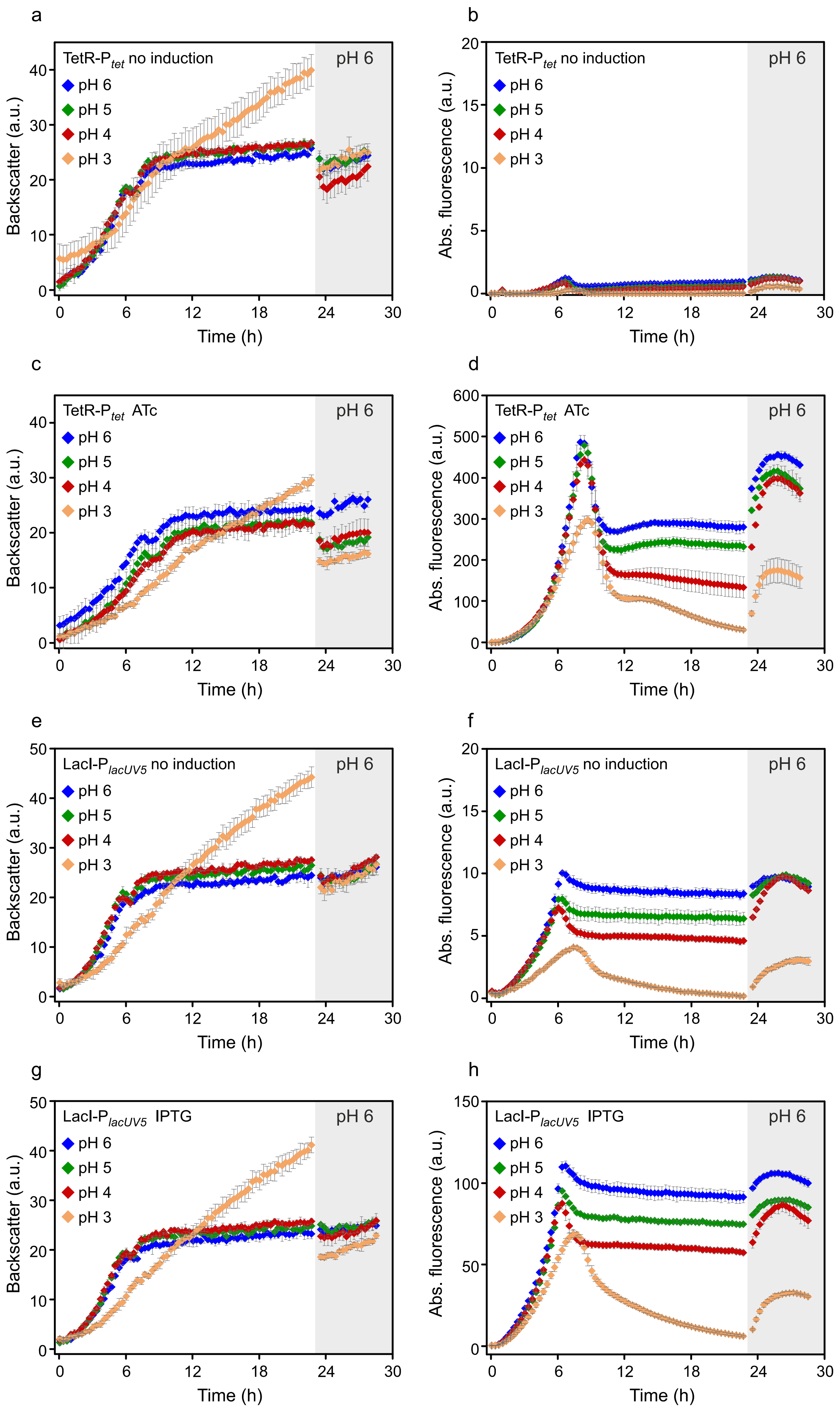
a
TetR-P_{tet} no induction
pH 6
pH 6
pH 5
pH 4
pH 3
Backscatter (a.u.)
Time (h)
b
TetR-P_{tet} no induction
pH 6
pH 6
pH 5
pH 4
pH 3
Abs. fluorescence (a.u.)
Time (h)
c
TetR-P_{tet} ATc
pH 6
pH 6
pH 5
pH 4
pH 3
Backscatter (a.u.)
Time (h)
d
TetR-P_{tet} ATc
pH 6
pH 6
pH 5
pH 4
pH 3
Abs. fluorescence (a.u.)
Time (h)
e
LacI-P_{lacUV5} no induction
pH 6
pH 6
pH 5
pH 4
pH 3
Backscatter (a.u.)
Time (h)
f
LacI-P_{lacUV5} no induction
pH 6
pH 6
pH 5
pH 4
pH 3
Abs. fluorescence (a.u.)
Time (h)
g
LacI-P_{lacUV5} IPTG
pH 6
pH 6
pH 5
pH 4
pH 3
Backscatter (a.u.)
Time (h)
h
LacI-P_{lacUV5} IPTG
pH 6
pH 6
pH 5
pH 4
pH 3
Abs. fluorescence (a.u.)
Time (h)

Fig. S3 Influence of the initial medium pH on the repression of P_{tet} and P_{lacUV5}.

Microscale BioLector cultivations with growth according to backscatter (**a, c, e, g**) and basal non-induced (**b, f**) and induced (**d, h**) expression of mNG according the absolute fluorescence of *G. oxydans* cells carrying either plasmid pBBR1MCS-5-T_{gdhM}-*tetR*-P_{tet}-*mNG*-T_{BBa_B1002}-T_{0028} or pBBR1MCS-5-P_{lacI}-*lacI*-P_{lacUV5}-RBS-*mNG*-T_{BBa_B1002}-T_{0028} grown in D-mannitol medium initially set to pH 6, 5, 4, and 3, respectively. After 23 h, all cells from each pH condition were transferred into fresh D-mannitol-free medium adjusted to pH 6 and again monitored in the BioLector (gray area, pH 6) to detect a potential pH-dependent recovery of mNG fluorescence signals above the respective levels monitored before. Data represent mean values and standard deviation from two biological replicates with three technical replicates each. Backscatter gain 15; fluorescence gain 50.

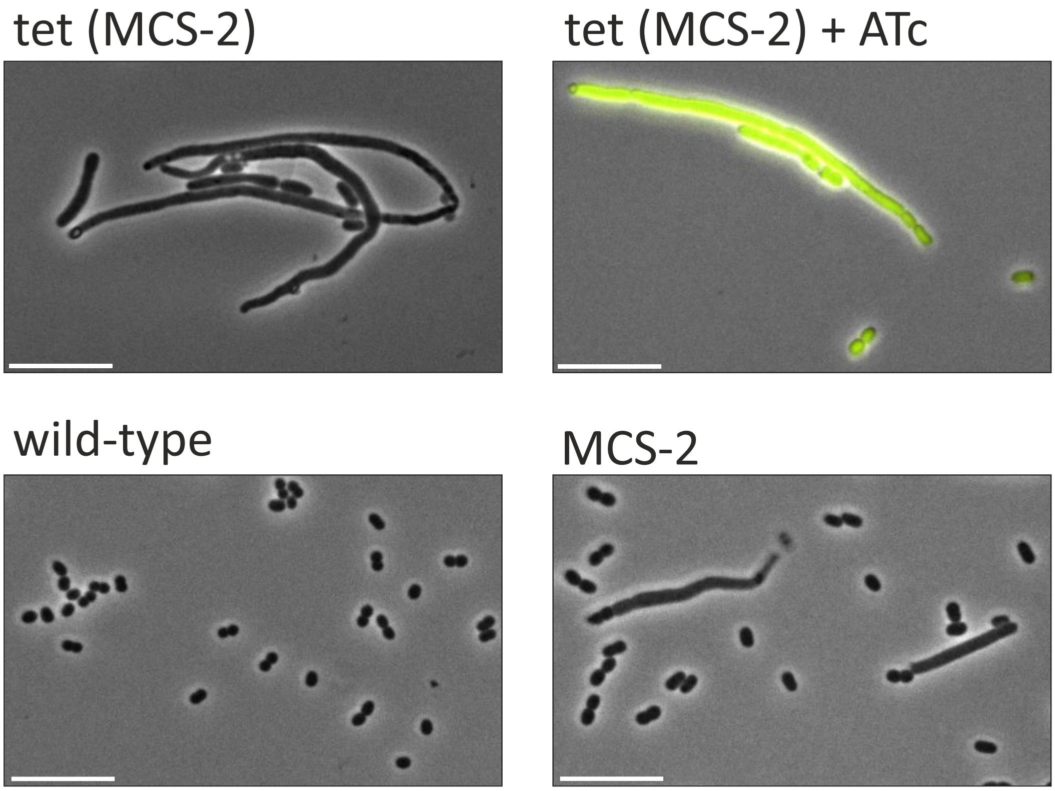

Fig. S4 Fluorescence microscopy of *G. oxydans* cells with pBBR1MCS-2 derivative.

Representative merged images (bright field + fluorescence channel) of *G. oxydans* 621H cells harboring plasmid pBBR1MCS-2-T_{gdhM}-*tetR*-P_{tet}-*mNG*-T_{BBa_B1002}-T_{0028} in anhydrotetracycline (ATc)-induced and non-induced condition ~7 h after inoculation (upper panel). Cells were grown in shake flasks in D-mannitol medium with 200 ng mL^{-1} ATc. As a control, *G. oxydans* 621H type strain without (wild-type) and with plasmid pBBR1MCS-2 were used (lower panel). Size bars represent 10 µm.

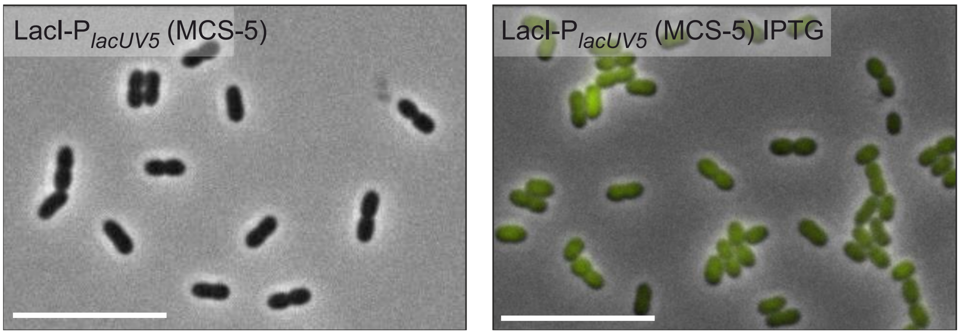

Fig. S5 Fluorescence microscopy of *G. oxydans* cells with LacI-P_{lacUV5} system.

Representative merged images (bright field + fluorescence channel) of *G. oxydans* 621H cells harboring plasmid pBBR1MCS-5-P_{lacI}-*lacI*-P_{lacUV5}-RBS-*mNG*-T_{BBa_B1002}-T_{0028} in non-induced and IPTG-induced condition ~7 h after inoculation and induction. Cells were grown in shake flasks without IPTG (left) and with 1 mM IPTG (right) upon inoculation. Size bars represent 10 µm. Images were taken on a Zeiss AxioImager M2 imaging microscope using the 46 HE filter set (λ_{ex} 500/20 nm / λ_{em} 535/30 nm).

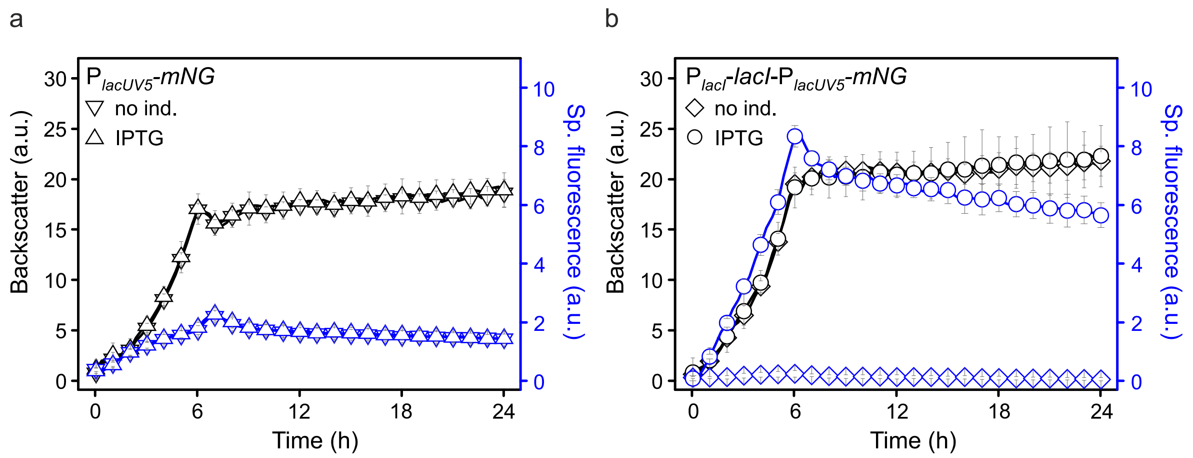

Fig. S6 Expression from P_{lacUV5} in the absence of the *lacI* gene.

Inducibility and expression strength of P_{lacUV5} in the absence and presence of P_{lacI}-*lacI* in microscale BioLector cultivations. The graphs show growth according to backscatter and biomass-specific mNG fluorescence in *G. oxydans* 621H carrying either plasmid pBBR1MCS-5-P_{lacUV5}-RBS-*mNG*-T_{BBa_B1002}-T_{0028} (**a**) or pBBR1MCS-5-P_{lacI}-*lacI*-P_{lacUV5}-RBS-*mNG*-T_{BBa_B1002}-T_{0028} (**b**) in IPTG-supplemented and non-supplemented condition. The specific fluorescence was calculated from absolute fluorescence per backscatter. For induction, always 1 mM IPTG was added to the D-mannitol medium. Data represent mean values and standard deviation from three biological replicates with three technical replicates each. Backscatter gain 15; fluorescence gain 60.

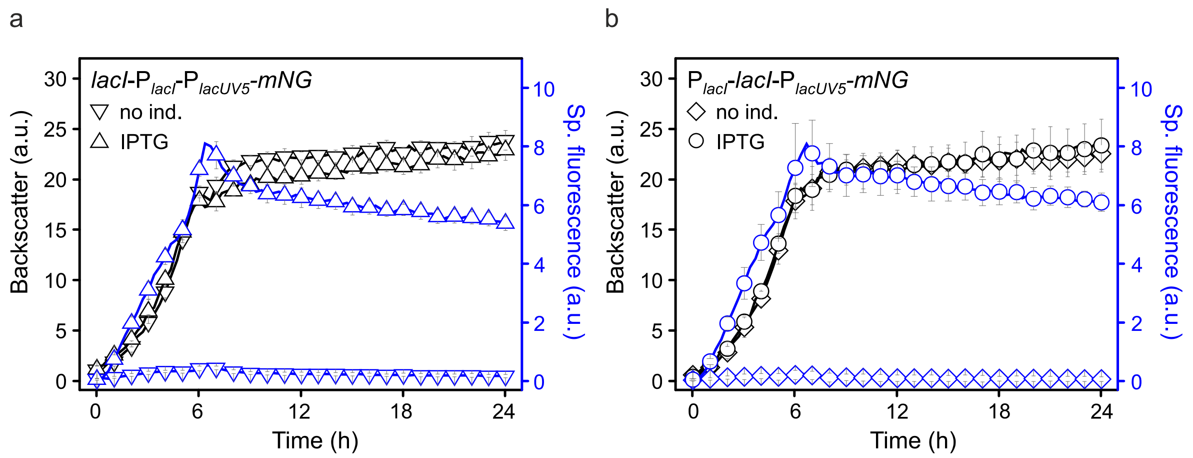

Fig. S7 Expression from $P_{\textit{lacUV5}}$ with opposite orientation of $P_{\textit{lacI}}$-*lacI*.

Inducibility and expression strength of $P_{\textit{lacUV5}}$ with $P_{\textit{lacI}}$-*lacI* in opposite orientation toward $P_{\textit{lacUV5}}$ (**a**) and in original arrangement (**b**) in microscale BioLector cultivations. The graphs show growth according to backscatter and biomass-specific mNG fluorescence in *G. oxydans* 621H carrying either plasmid pBBR1MCS-5-*lacI*-$P_{\textit{lacI}}$-$P_{\textit{lacUV5}}$-RBS-*mNG*-T_{BBa_B1002}-T_{0028} (**a**) or pBBR1MCS-5-$P_{\textit{lacI}}$-*lacI*-$P_{\textit{lacUV5}}$-RBS-*mNG*-T_{BBa_B1002}-T_{0028} (**b**) in IPTG-supplemented and non-supplemented condition. The specific fluorescence was calculated from absolute fluorescence per backscatter. For induction, always 1 mM IPTG was added to the D-mannitol medium. Data represent mean values and standard deviation from at least three biological replicates with three technical replicates each. Backscatter gain 15; fluorescence gain 60.

Text S1 Generation and evaluation of TetR, LacI, and AraC homology models

The three dimensional structures of TetR, LacI, and AraC were constructed by YASARA (Krieger et al. 2002). Fig. S8-S10 show the modelled structures. The hybrid homology models of TetR, LacI, and AraC were evaluated with the verify3D and ProSA-web webservers (Sippl 1993). Detailed results are described in the following.

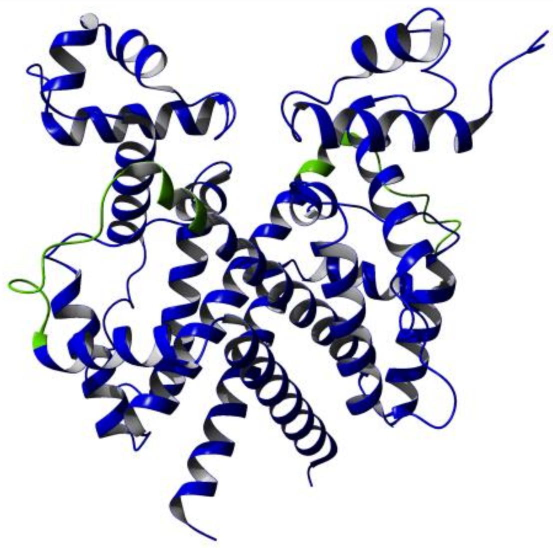

Fig. S8 3D structure of TetR modelled with the Homology Modelling function of YASARA (Z score: 0.480). The model is a hybrid model based on the crystal structures with PDB ID 3ZQI and 2XPW. Residues contributed by 3ZQI (1-152 and 169-208) are visualized in blue and residues contributed by 2XPW (152-168) in green.

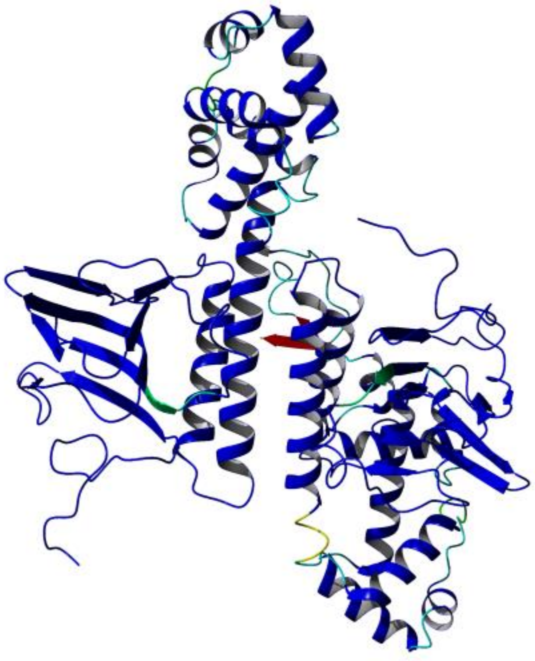

Fig. S9 3D structure of AraC modelled with YASARA (Z score: -0.229), a hybrid model based on crystal structures with PDB ID 2AAC, 2K9S, and 1XJA.

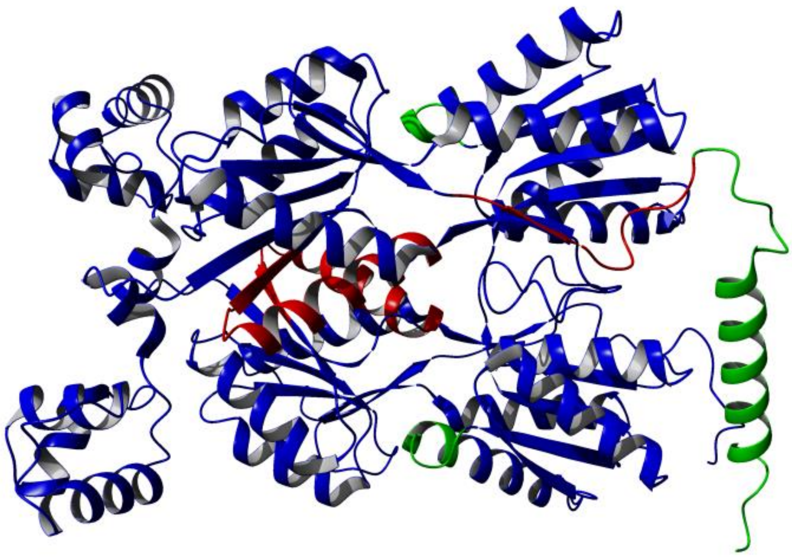

Fig. S10 LacI hybrid homology model modelled with YASARA (Z score: -0.143) based on crystal structures with PDB ID 1EFA (blue) and 2EDC (red and green).

Text S1.1 Evaluation of the TetR model

Verify3D analysis of TetR model showed that only 74.5% of the residues of the hybrid homology model of TetR have an average 3D/1D score above 0.2 (Fig S11). That indicated that the three-dimensional structure of the model was not fully compatible with the amino acid sequence. However, the Z score of the model was calculated to be -7.27 according to the ProSA-web server and lies within the range of scores typically found for experimentally determined structures (Fig. S12, left). The local quality score of the TetR model lies below 0.0 for a window size of 40 residues (Fig. S12, right), indicating that there are no problematic or erroneous parts in the model.

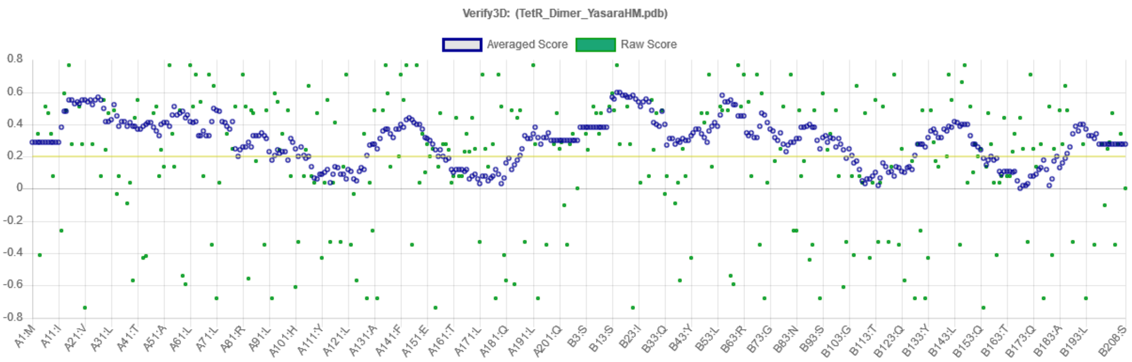

Fig. S11 Verify3D analysis of the TetR hybrid homology model. Average (blue) and raw (green) 3D/1D scores. Values above 0.2 indicate that the 3D structure is compatible with the amino acid sequence of the protein.

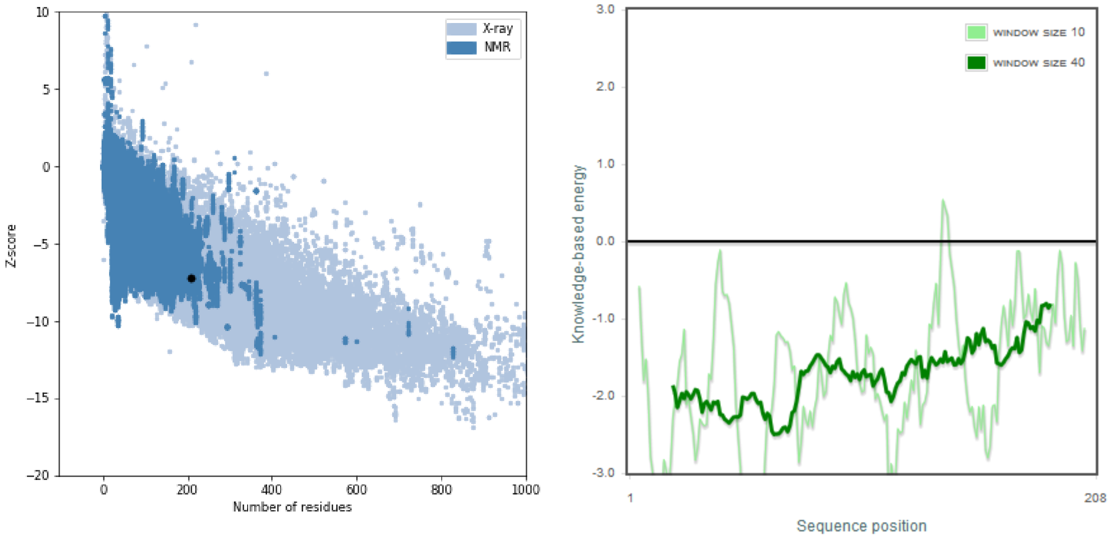

Fig. S12 ProSA-web server results for the TetR hybrid homology model. Left: Global quality of the TetR hybrid homology model. The black dot indicates the model quality in relation to the score of experimentally determined structures (blue). Right: Local model quality of the TetR hybrid homology model calculated from the knowledge-based energy within a 40 (dark green) or 10 (light green) residues window.

Text S1.2 Evaluation of the AraC hybrid homology model

Verify3D analysis of the AraC hybrid homology model suggested that its three-dimensional structure correlated well with its amino acid sequence. For 83.5% of the residues the 3D/1D score was above 0.2 (Fig. S13). The Z score -7.31 of the model calculated with ProSA-web was in the range of scores of experimentally determined protein structures (Fig. S14, left). The local quality score displayed higher values at N- and C-terminal residues of the protein, indicating that the model quality is lower at these positions (Fig. S14, right).

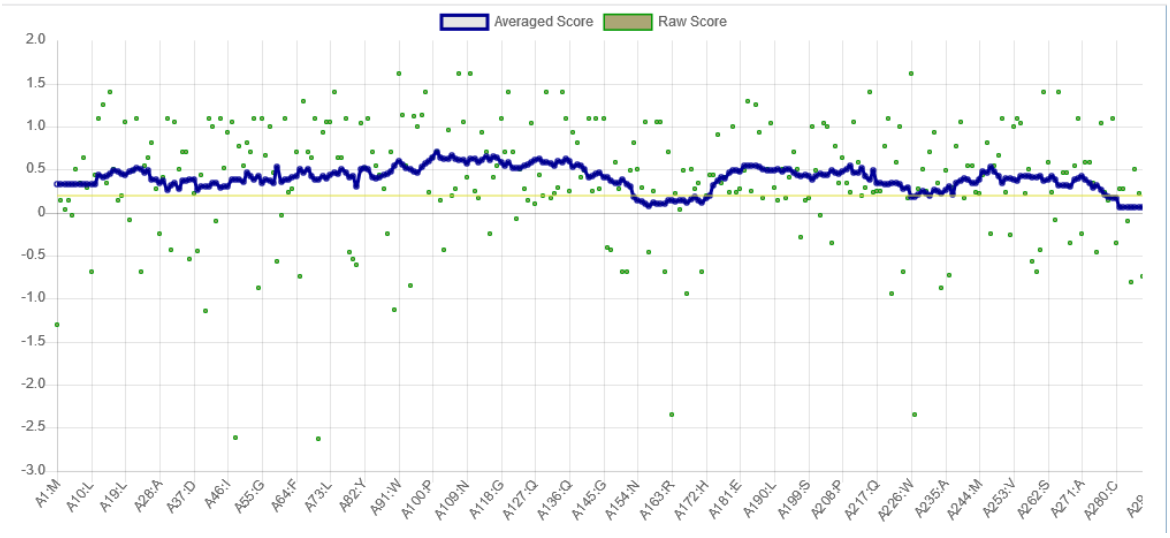

Fig. S13 Verify3D analysis of AraC hybrid homology model. Average (blue) and raw (green) 3D/1D scores. Values above 0.2 indicate that the 3D structure is compatible with the amino acid sequence.

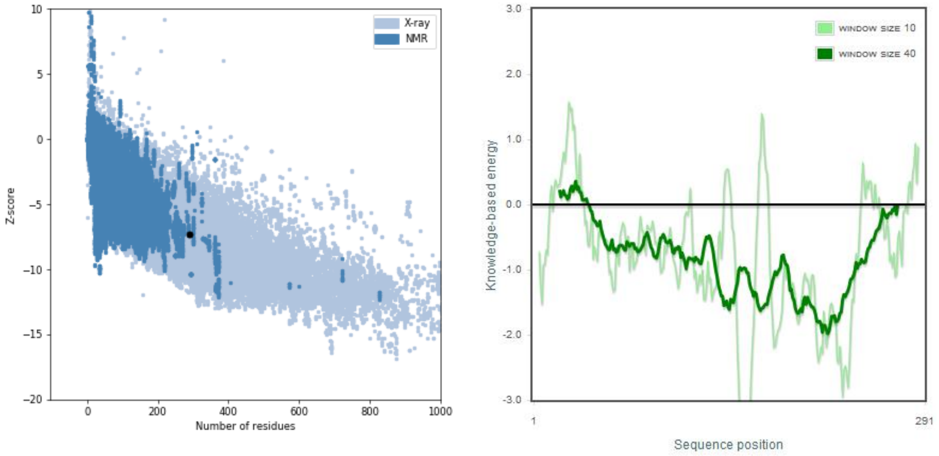

Fig. S14 ProSA-web results for AraC hybrid homology model. Left: Global quality of the AraC hybrid homology model. The black dot indicates the model quality in relation to the score of experimentally determined structures (blue). Right: Local quality of the AraC hybrid homology model calculated from the knowledge-based energy within a 40 (dark green) or 10 (light green) residues window.

Text S1.3 Evaluation of the LacI hybrid homology model

Verify3D analysis of LacI showed that 89.7% of the residues of the hybrid homology model have an average 3D/1D score above 0.2 (Fig. S15). Therefore it could be assumed that the 3D structure of the model was compatible with its amino acid sequence. The 3D structure was also evaluated with ProSA-web and a Z score of -8.41 was obtained. This Z score was within the range of scores calculated for experimentally determined protein structures (Fig. S16, left). Most residues of the LacI model have a local quality score below 0.0 (Fig. S16, right). This indicated that most parts of the model are unproblematic.

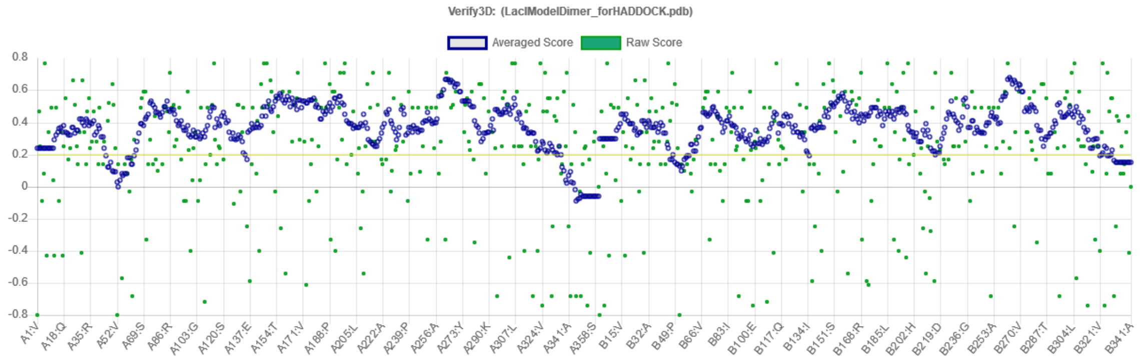

Fig. S15 Verify3D analysis of the LacI hybrid homology model. Average (blue) and raw (green) 3D/1D scores. Values above 0.2 indicate that 3D structure is compatible with the amino acid sequence.

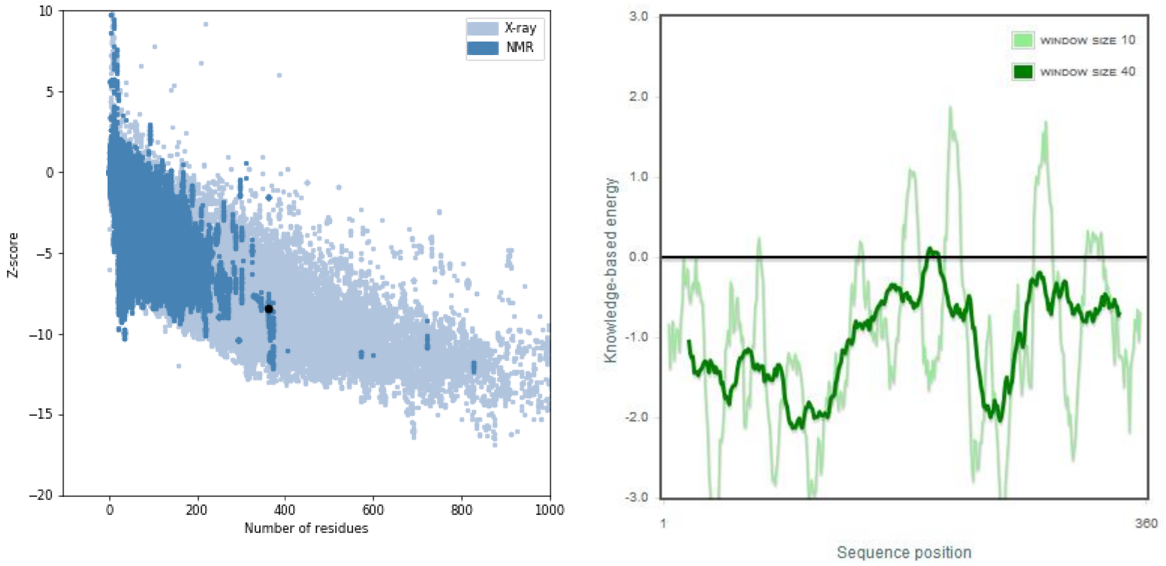

Fig. S16 ProSA-web results for LacI hybrid homology model. Left: Global quality of the LacI hybrid homology model. The black dot indicates the model quality in relation to the score of experimentally determined structures (blue). Right: Local quality of the LacI hybrid homology model calculated from the knowledge-based energy within a 40 (dark green) or 10 (light green) residues window.

Text S2 Protein-DNA and protein-protein docking simulations with HADDOCK webserver

The DNA binding and oligomerization behaviour at different pH values were investigated with the HADDOCK webserver (van Zundert et al. 2016). The docking behaviour of all transcription factors to DNA was investigated for pH 7, 6, 5, and 4 by submitting the transcriptional factor models with their respective DNA structures to the HADDOCK webserver. For TetR the full protein model was used, while for AraC a monomer of the hybrid homology model and for LacI a dimer model were used, respectively.

The oligomerization behaviour (probable quaternary structures) was investigated by submitting the relevant subunits of the transcription factors as individual objects to the run. For TetR and AraC both monomers of the YASARA hybrid models were used as input files for HADDOCK calculations. Due to a lacking tetramerization helix, the YASARA hybrid model of LacI was not suitable for this analysis. Instead, the different dimers of the crystal structure with PDB ID 3EDC were submitted to the HADDOCK webserver. For all transcription factors pH 7, 6, 5, and 4 were analysed.

The HADDOCK webserver models several structures for each docking run and clusters them. For each cluster a score is calculated that contains intermolecular energies including Van-der-Waals energy, electrostatic energies, desolvation energy, and distance restraints energy. This score were used to determine the best docking position and to compare the binding behaviour at the different pH values.

To further evaluate the different clusters, the best structure of the best-scoring clusters were visually compared to each other and, if available, to the crystal structure of the respective transcription factor with its respective DNA operator fragment. This was especially important for clusters that displayed HADDOCK scores within the standard deviation of each other. Sizes and scores of the five best-scoring clusters of each docking simulation were summarized (Table S2).

Table S2 HADDOCK webserver results for protein-DNA and protein-protein docking of the transcriptional factors TetR, AraC and LacI to their respective operator DNA at different pH values. HADDOCK scores that lie within each other's standard deviation are displayed in bold.

HADDOCK docking	pH7			pH6			pH5			pH4		
	Cluster	Size	Score	Cluster	Size	Score	Cluster	Size	Score	Cluster	Size	Score
DNA-TetR	6	8	**-89.3±9.9**	2	17	**-86.3±2.7**	1	19	**-82.8±3.6**	1	20	**-99.0±3.8**
	1	17	**-83.9±5.7**	1	19	**-86.1±3.1**	4	12	**-78.9±6.6**	4	10	**-90.4±7.2**
	2	16	**-80.7±5.4**	3	15	**-84.5±2.9**	2	19	**-77.0±2.5**	8	5	**-85.2±3.8**
	3	12	**-78.4±3.8**	6	7	**-75.8±11.2**	9	7	**-76.4±6.6**	15	4	**-84.1±15.6**
	4	12	**-74.0±3.8**	8	6	**-73.2±4.6**	3	12	**-76.3±6.8**	3	12	**-83.2±7.1**
DNA-AraC	1	28	**-72.8±4.1**	13	5	**-98.3±10.3**	13	5	**-94.8±12.8**	1	24	**-94.4±2.4**
	8	6	**-66.5±5.1**	1	32	**-91.3±7.9**	4	15	**-86.1±5.8**	2	19	-86.4±1.2
	2	17	**-64.1±4.6**	11	6	**-78.9±16.3**	1	19	**-77.0±8.8**	5	13	-82.2±5.6
	3	16	**-58.3±2.7**	2	17	**-76.8±0.7**	2	17	**-75.0±8.6**	14	4	-77.7±12.4
	5	12	**-56.8±4.4**	10	6	**-76.6±8.1**	8	7	**-75.0±10.8**	4	14	-77.6±17.2
DNA-LacI	**4**	**9**	**-128.2±13.0**	**4**	**6**	**-112.5±10.1**	**12**	**4**	**-106.0±6.7**	**9**	**4**	**-102.1±8.3**
	3	9	-88.7±4.7	1	13	-84.5±7.7	1	16	-87.4±6.3	1	17	-86.2±3.4
	8	6	-82.5±11	3	8	-75.9±4.4	11	4	-79.2±7.3	5	6	-85.9±9.1
	1	14	-82.0±2.1	10	4	-65.9±6.8	4	6	-78.3±6.3	4	6	-75.2±10.8
	6	6	-74.6±12.7	2	9	-64.2±10.4	2	14	-77.9±7.1	2	9	-67.4±7.4

TetR dimerization	**7**	**4**	**-330.4±45.4**	**7**	**5**	**-126.4±20.1**	3	43	**-91.8±4.4**	5	9	**-96.0±16.8**
	6	11	-91.8±6.2	2	39	-88.5±2.1	6	6	**-90.4±11.1**	1	57	**-89.8±3.1**
	1	65	-76.6±3.0	1	53	-82.8±2.5	1	47	**-78.5±3.1**	4	27	**-85.6±9.9**
	3	26	-75.6±5.6	3	36	-80.0±1.4	4	16	**-75.5±1.6**	2	48	**-81.4±3.5**
	2	37	-73.8±4.2	4	23	-72.2±3.9	2	45	**-73.9±4.1**	3	27	**-73.9±4.4**
AraC dimerization	6	10	**-127.2±8.0**	1	22	**-149.6±7.6**	5	16	**-133.5±4.6**	1	27	**-134.2±6.7**
	2	16	-108.5±2.8	3	15	**-137.9±10.5**	3	18	**-132.3±8.0**	3	16	**-125.5±12.2**
	10	7	-108.0±6.0	14	6	-110.7±8.4	4	17	-109.2±9.0	2	17	-102.2±4.8
	1	18	-105.1±6.8	2	16	-109.7±4.0	1	20	-108.5±7.0	7	8	-99.6±14.0
	4	11	-99.3±4.6	7	9	-106.3±9.7	2	18	-106.6±5.2	8	8	-92.5±8.5
LacI tetramerization	**2**	**39**	**-223.3±11.7**	**2**	**29**	**-206.8±1.4**	**2**	**31**	**-203.0±3.9**	**2**	**40**	**-187.5±3.3**
	1	55	-111.3±8.5	1	54	-97.3±6.8	1	62	-87.0±6.3	1	53	-60.6±9.9
	9	7	-76.3±26.1	8	7	-93.3±5.3	4	14	-70.2±17	8	5	-45.8±36.2
	5	11	-60.3±13.5	4	16	-54.0±12.2	8	6	-35.7±6.4	3	17	-43.7±15.4
	4	11	-57.1±3.5	5	11	-47.5±15.2	3	17	-35.0±2.9	5	10	-39.2±8.6

Text S2.1 DNA binding and dimerization behaviour of TetR at different pH values

For the docking simulation of TetR to its DNA operator sequence, the whole protein model obtained from YASARA was used. The HADDOCK scores of all five best-scoring clusters at pH 7, 6, 5 and 4 were within standard deviation of each other (Table S2). However, without any further information on which clusters contain biological valid docking positions, a reliable assumption about the docking behaviour for different pH values could not be made. Therefore, a visual comparison of the first structure of each cluster to the crystal structure with PDB ID 1QPI was conducted. The crystal structure 1QPI consists of a TetR monomer bound to a single stranded DNA fragment, and can therefore only be taken as an approximation of the native docking position.

The TetR alignments at pH 7, 6, 5, and 4 were visualized (Fig. S17-S20). It became clear that none of the docking positions modelled with HADDOCK correlated with the crystal structure 1QPI. However, some docking positions could be identified in clusters of each run. The docking position of cluster 1 at pH 7 (Fig. S17, B) correlated with that of cluster 2 at pH 6 (Fig. S18, A) and cluster 1 at pH 4 (Fig. S20, A). For pH 7 (Fig. S17, E), pH 5 (Fig. S19, B) and pH 4 (Fig. S20, B) the clusters 4 aligned as well. These two groups of clusters were used to depict the binding behaviour for the investigated pH shift (Fig. S21).

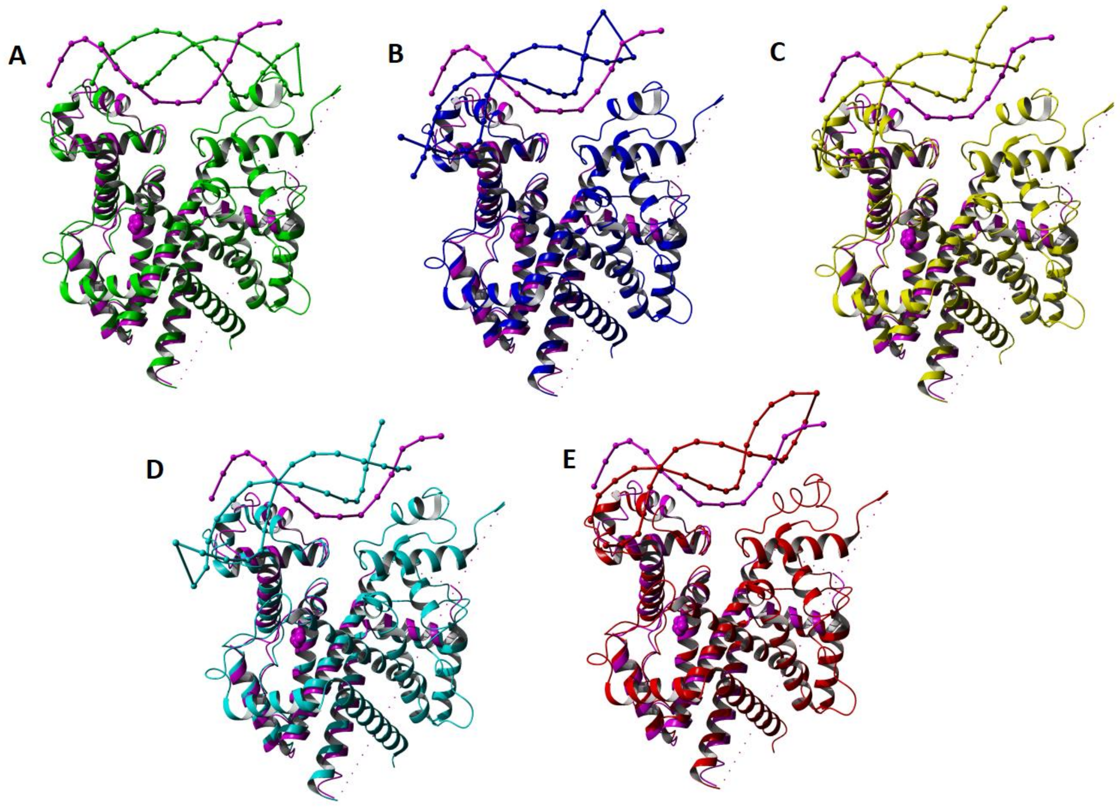

Fig. S17 DNA-protein docking results for TetR at pH 7. The first structure of cluster 6 (**A**, green), cluster 1 (**B**, blue), cluster 2 (**C**, yellow), cluster 3 (**D**, cyan), and cluster 4 (**E**, red) aligned to 1QPI (magenta).

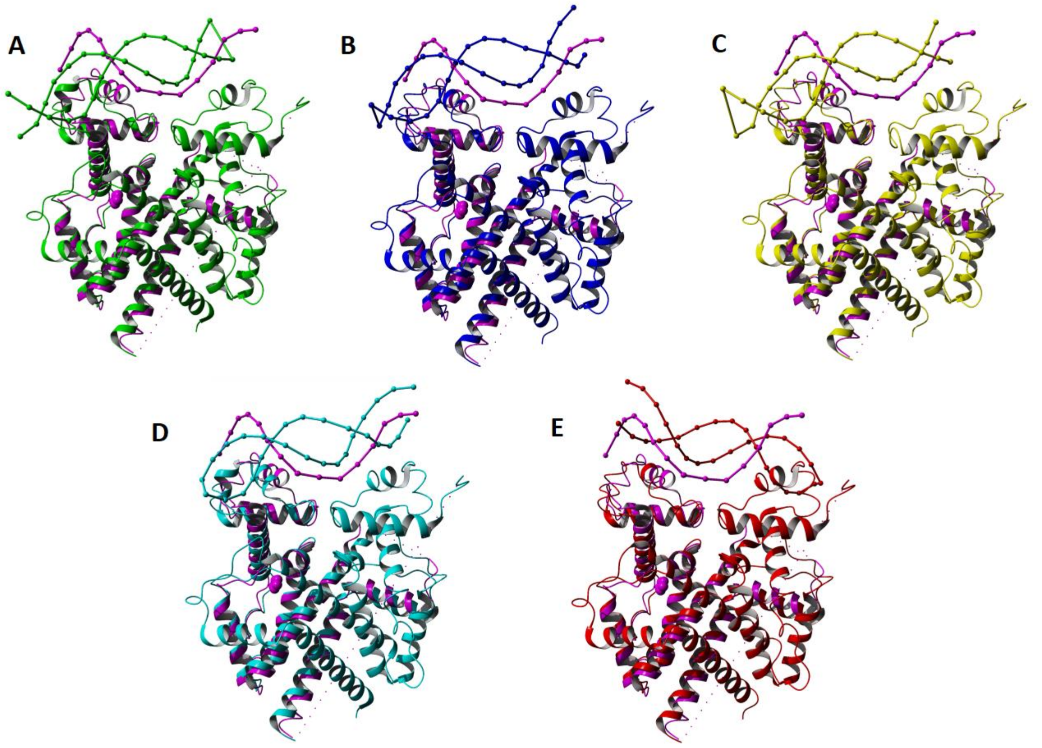

Fig. S18 DNA-protein docking results for TetR at pH 6. The first structure of cluster 2 (**A**, green), cluster 1 (**B**, blue), cluster 3 (**C**, yellow), cluster 6 (**D**, cyan), and cluster 8 (**E**, red) aligned to 1QPI (magenta).

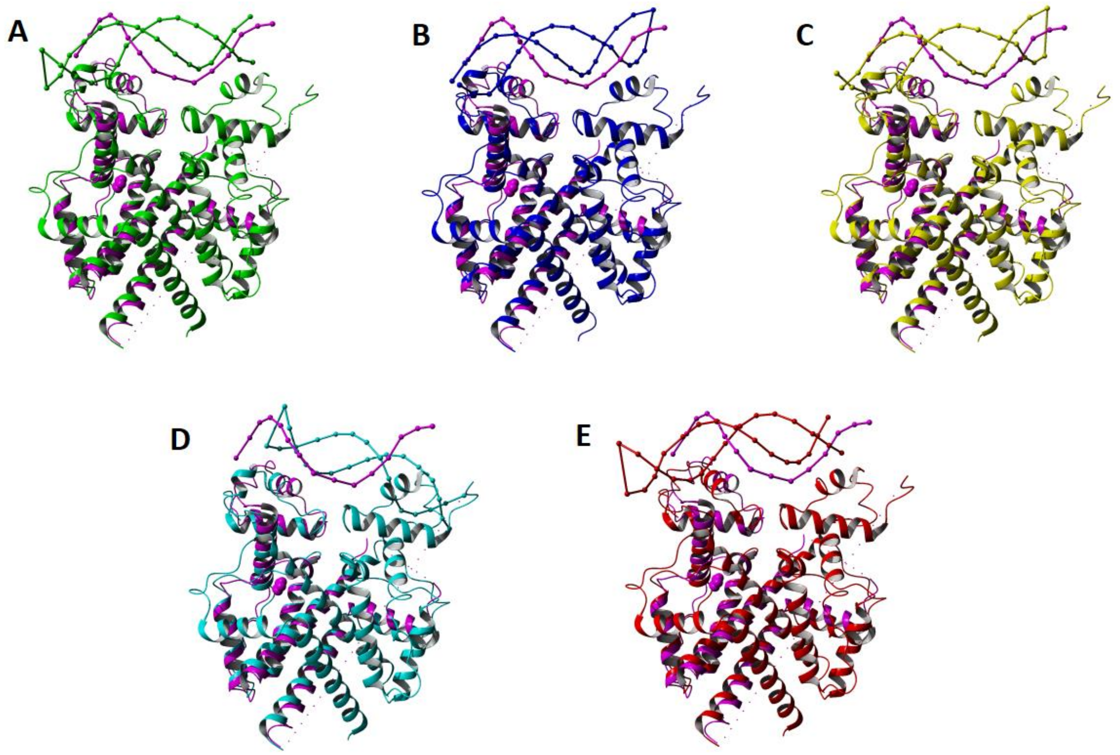

Fig. S19 DNA-protein docking results for TetR at pH 5. The first structure of cluster 1 (**A**, green), cluster 4 (**B**, blue), cluster 2 (**C**, yellow), cluster 9 (**D**, cyan), and cluster 3 (**E**, red) aligned to 1QPI (magenta).

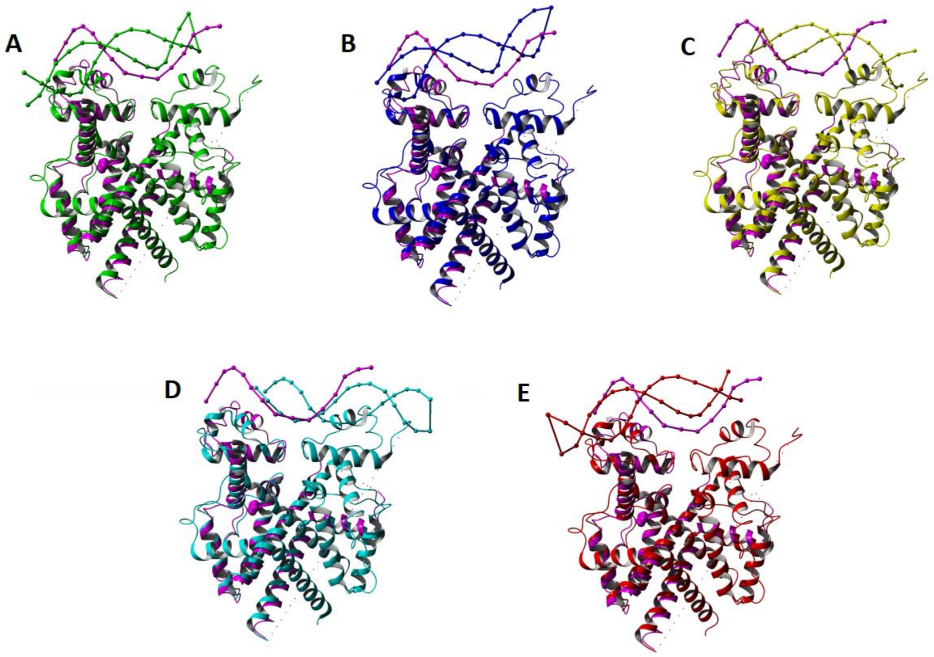

Fig. S20 DNA-protein docking results for TetR at pH 4. The first structure of cluster 1 (**A**, green), cluster 4 (**B**, blue), cluster 8 (**C**, yellow), cluster 15 (**D**, cyan), and cluster 3 (**E**, red) aligned to 1QPI (magenta).

Both cluster groups displayed decreasing HADDOCK scores when pH values decreased (Fig. S21). Assuming that the selected docking positions are representative for the native docking position, the scores suggested that DNA binding of TetR increases with a decreasing pH with a higher rate of change for lower pH values. A more precise prediction would require data for basic conditions. For pH 7 and 6 it seems that DNA binding could remain unaltered for circumneutral pH values and begins to increase only for pH values lower than six according to the trend line based on both cluster groups (Fig. S21, dark blue).

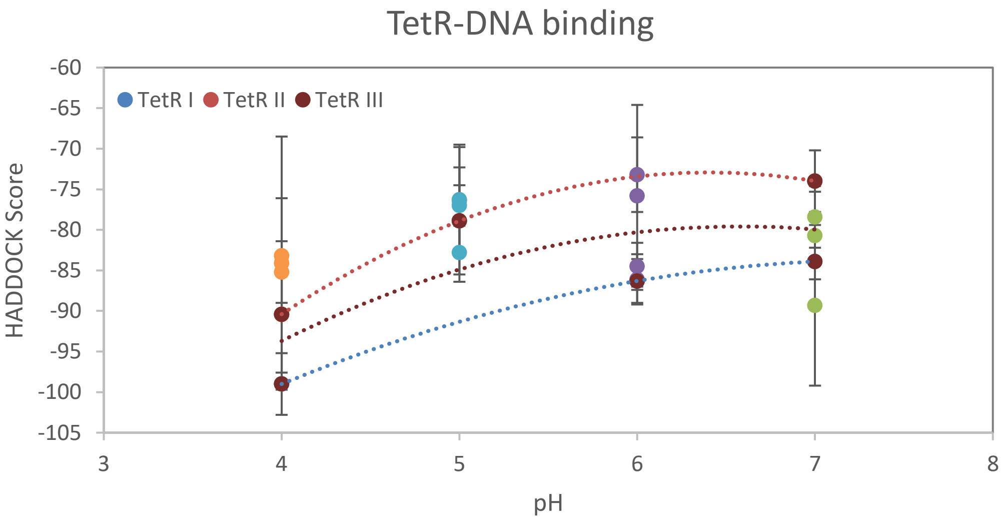

Fig. S21 TetR-DNA binding behaviour at different pH computed with the HADDOCK webserver. HADDOCK scores of all clusters versus the pH values. Trend line TetR I is based on cluster 1 (pH 7), cluster 2 (pH 6), and cluster 1 (pH 4). Trend line TetR II is based on the clusters 4 at pH 7, 5, and 4. Trend line TetR III is based on both cluster groups. A second-degree polynomial fitting was used for all trend lines.

For the dimerization simulation, the monomers of the YASARA hybrid model were submitted to the HADDOCK webserver as individual binding partners. The best-scoring cluster for pH 7, e.g. cluster 7, is also the only structure that aligns perfectly with the initial YASARA hybrid homology model (Fig. S22-S25). In contrast to the DNA binding simulation, the dimerization simulation showed very clearly different results for pH 7 and pH 6. For pH 7 a HADDOCK score of -330.4 ± 45.4 was obtained, and a score of -220.8 ± 7.9 for pH 6. Despite the high standard deviation, the HADDOCK score of the best-scoring cluster for pH 7 did not overlap neither with other high-scoring clusters for pH 7, nor with the clusters for pH 6, 5 or 4 (Fig. S26).

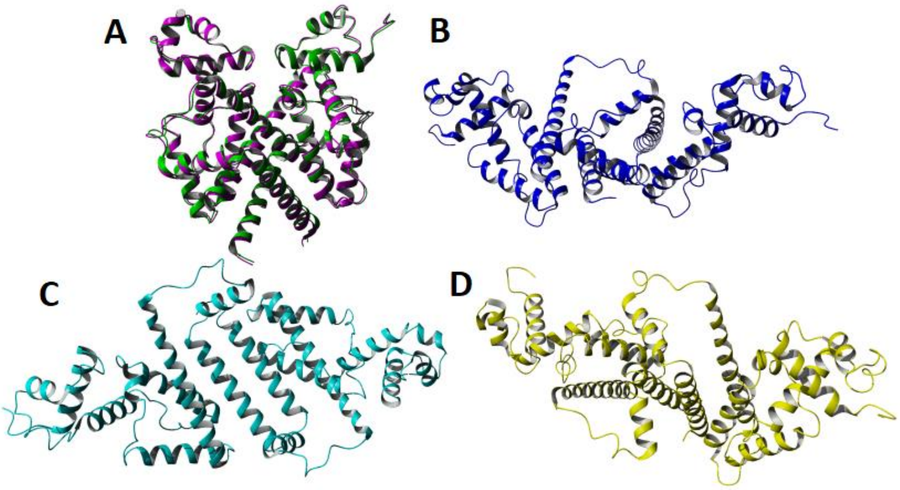

Fig. S22 TetR dimerization results of the HADDOCK webserver at pH 7. **A**: Alignment of the TetR YASARA hybrid model (magenta) and cluster 7 (green), **B**: cluster 6 (blue), **C**: cluster 3 (cyan), **D**: cluster 1 (yellow).

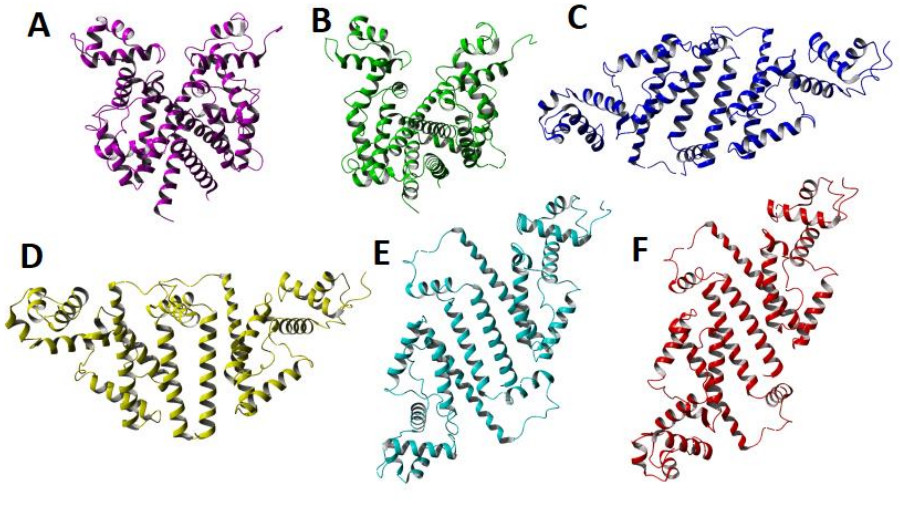

Fig. S23 TetR dimerization results of the HADDOCK webserver at pH 6. **A**: The TetR YASARA hybrid model (magenta), **B**: cluster 7 (green), **C**: cluster 2 (blue), **D**: cluster 1 (yellow), **E**: cluster 3 (cyan), **F**: cluster 4 (red).

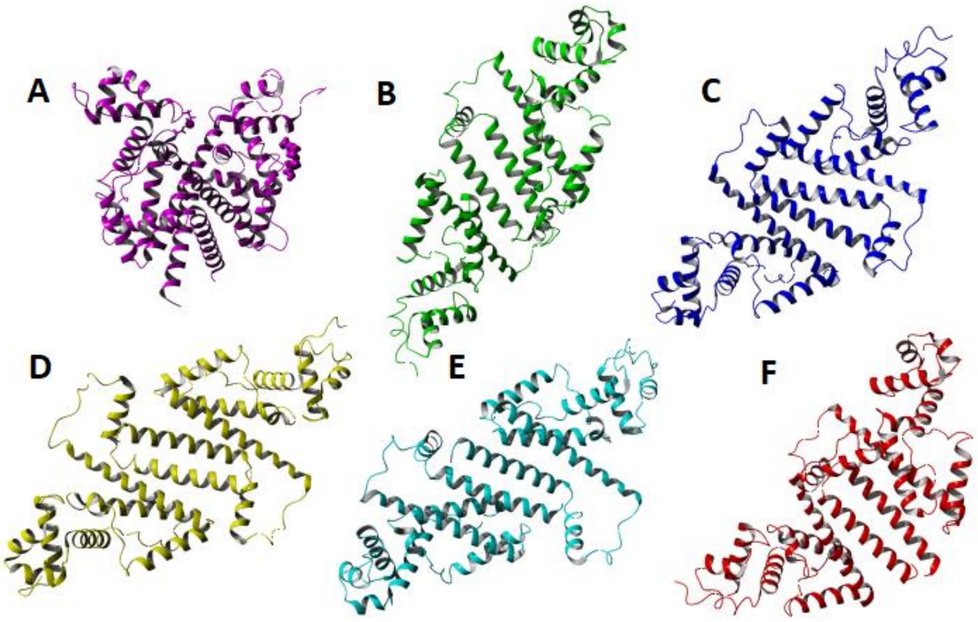

Fig. S24 TetR dimerization results of the HADDOCK webserver at pH 5. **A**: The TetR YASARA hybrid model (magenta), **B**: cluster 3 (green), **C**: cluster 6 (blue), **D**: cluster 1 (yellow), **E**: cluster 4 (cyan), **F**: cluster 2 (red).

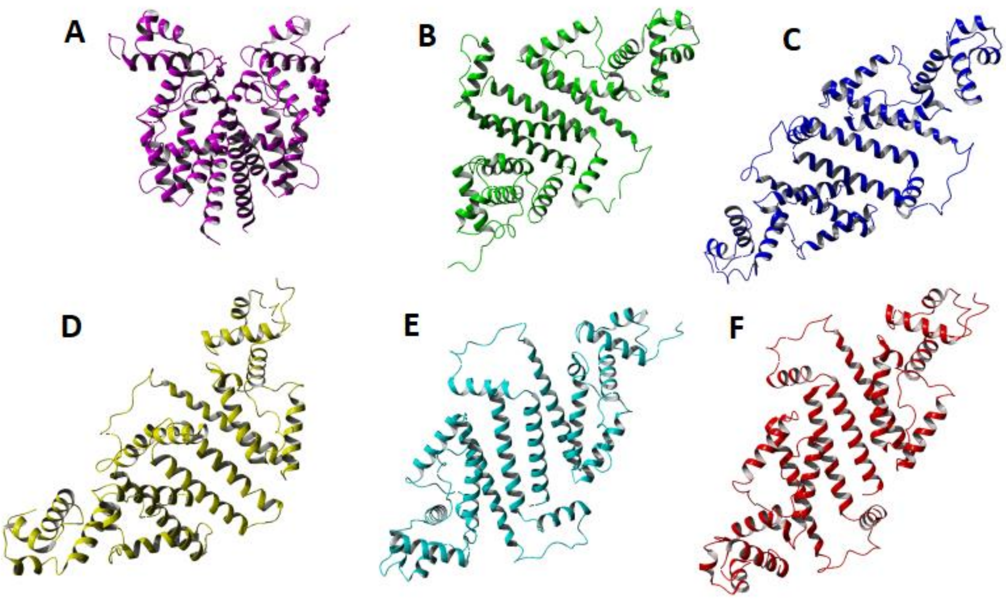

Fig. S25 TetR dimerization results of the HADDOCK webserver at pH 4. **A**: The TetR YASARA hybrid model (magenta), **B**: cluster 5 (green), **C**: cluster 1 (blue), **D**: cluster 4 (yellow), **E**: cluster 2 (cyan), **F**: cluster 3 (red).

Based on the significant difference between the HADDOCK scores at pH 7, 6, 5, and 4, and the fact, that the HADDOCK webserver was not able to model the correct dimerization state at pH 6, 5, and 4, it could be hypothesized that TetR dimerization may only occur sufficiently at circumneutral pH values. The trend line based on the best-scoring clusters, suggested that dimerization decreases rapidly between pH 7 and 6, and then remains almost unaltered for pH values lower than 5.5 (Fig. S26).

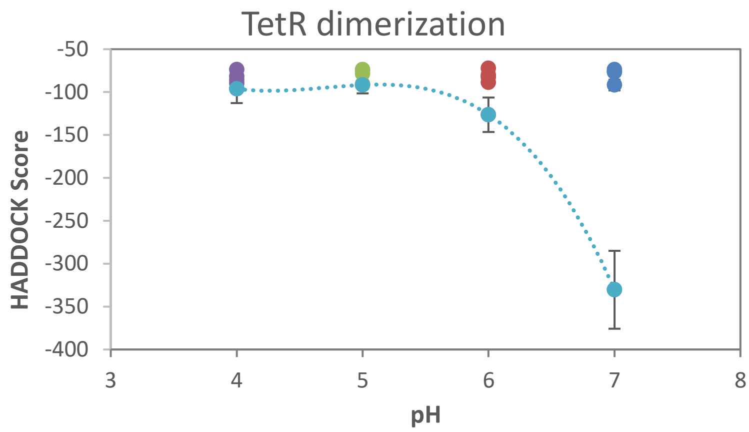

Fig. S26 TetR dimerization behaviour at different pH. HADDOCK scores of the five best-scoring clusters of each run versus the pH values. The blue trend line is based on the best-scoring cluster of each run. A third-degree polynomial fitting was used.

Text S2.2 DNA binding and dimerization behaviour of AraC at different pH values

For the docking simulation of AraC to its operator sequence, monomer A of the YASARA hybrid model was used. The HADDOCK scores of all five best-scoring clusters at pH 7, 6, and 5 were within the standard deviation of each other (Table S2). Only for pH 4 a unique cluster was modelled with a score of -94.4 ± 2.4. However, without a structure of AraC co-crystallized with its operator sequence, crucial information about the correct binding position is missing and the identification of the most reliable cluster based on the HADDOCK scores alone might be inaccurate. Therefore, the different modelled docking positions were aligned and clusters that contained a similar binding position were used as approximation to predict binding behaviour. Cluster 1 (pH 7), cluster 4 (pH 5), and cluster 1 (pH 4) aligned very well (Fig. S27). This alignment was used for the evaluation of the binding behaviour of AraC to DNA (Fig. S28). The trend line suggested, that DNA binding behaviour increases almost linearly with decreasing pH values.

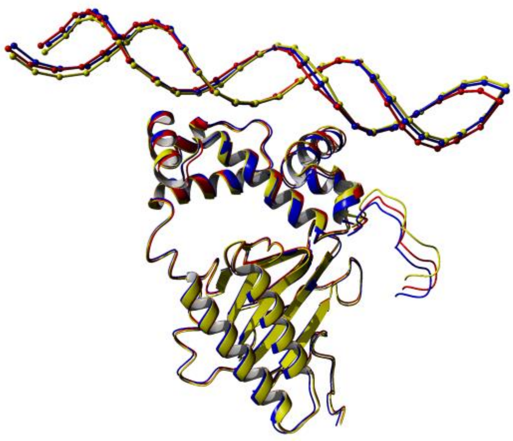

Fig. S27 Alignment of AraC-DNA docking positions of cluster 1 (pH 7, red), cluster 4 (pH 5, blue), and cluster 1 (pH 4, yellow).

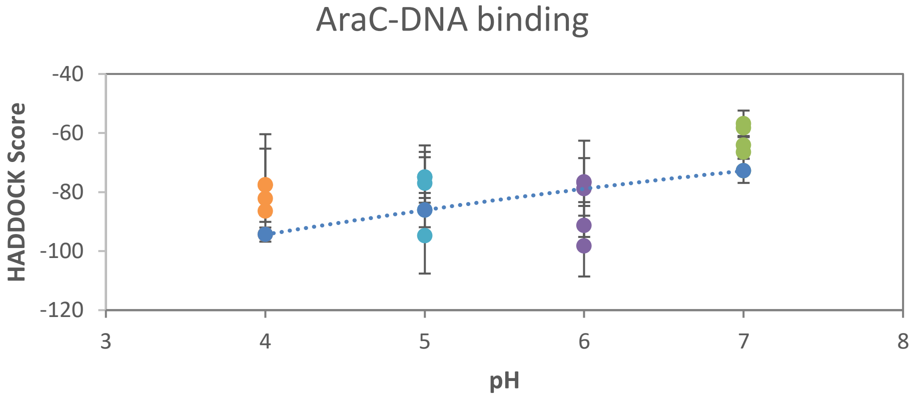

Fig. S28 AraC-DNA binding behaviour at different pH. HADDOCK scores of the five best-scoring clusters versus the pH values. The blue trend line is based on cluster 1 (pH 7), cluster 4 (pH 5), and cluster 1 (pH 4). A second-degree polynomial fitting was used.

For the dimerization modeling both AraC monomers were used as input for the HADDOCK webserver. In contrast to the DNA docking simulation, the dimerization modeling displayed much clearer results (Table S2). For pH 7, cluster 6 displayed the best HADDOCK score with a value of -127.2 ± 8.0. For pH 6, the best-scoring clusters were cluster 1 and 3 with scores of -149.6 ± 7.6 and -137.9 ± 10.5. For pH 5, the two best-scoring clusters 5 and 3 exhibited a score of -133.5 ± 4.6 and -132.3 ± 8.0, respectively. For pH 4, cluster 1 and 3 exhibited scores of -134.2 ± 6.7 and -125.5 ± 12.2. By taking only the best-scoring clusters into account, the scores suggested that AraC dimerization behaviour should not change significantly with decreasing pH value (Fig. S33).

Interestingly, when comparing the structures modelled with the HADDOCK webserver to the YASARA hybrid model, the best-scoring clusters do not align best with the initial model (Fig. S29-S32). In each case, the DNA binding domains of both monomers are oriented towards the same side and not to opposite sides of the protein core, while for lower-scoring clusters the structures displayed a symmetry that aligned much better with the structure of the initial model. For pH 6 and 5 an additional conformation was be observed, in which both DNA binding domains were also positioned on the same side of the protein core, but are much closer to each other (Fig. S30D, Fig. S31D).

It could be argued, that the different conformations correlate with the different states of the transcription factor, i.e. DNA binding region on opposite sides of the core protein correlates with the inducer-unbound state, DNA binding region on the same side of the core protein correlates with the inducer-bound state of the transcription factor. However, despite differences in the absolute values of the HADDOCK scores for the different conformations, the conclusion seems to be the same. For a pH value decreasing from pH 7, the dimerization behaviour of AraC is not affected (Fig. S33).

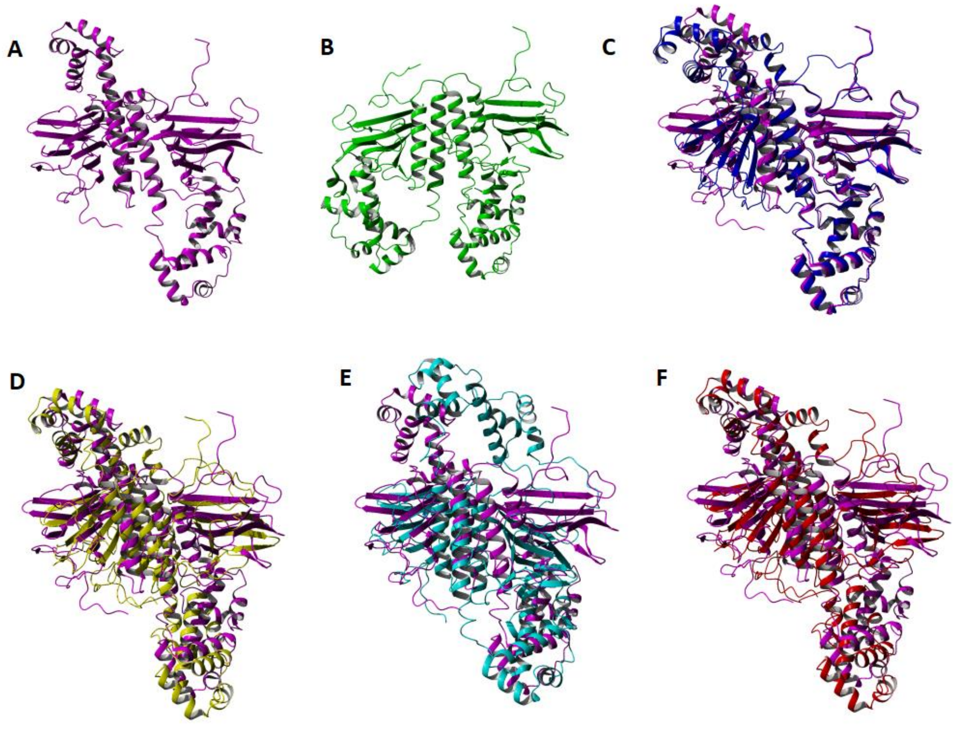

Fig. S29 AraC dimerization results of the HADDOCK webserver at pH 7. **A**: The YASARA AraC hybrid model (magenta), **B**: cluster 6 (green). Alignment between the AraC model and cluster 2 (**C**, blue), cluster 10 (**D**, yellow), cluster 1 (**E**, cyan) and cluster 4 (**F**, red).

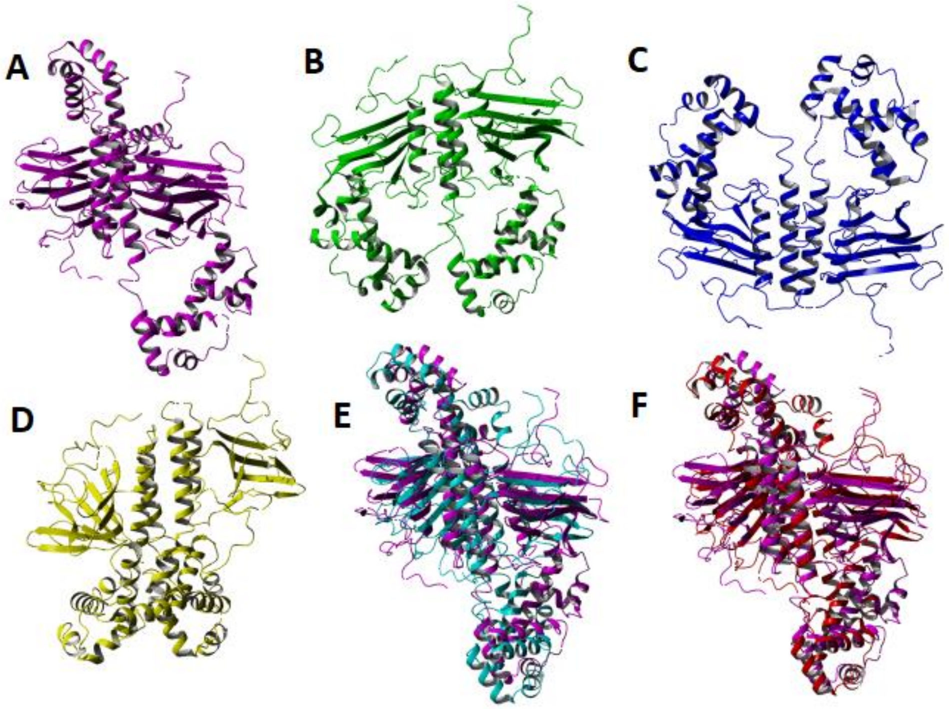

Fig. S30 AraC dimerization results of the HADDOCK webserver at pH 6. **A**: YASARA AraC hybrid model (magenta), **B**: cluster 1 (green), **C**: cluster 3 (blue), **D**: cluster 14 (yellow), **E**: Alignment between AraC model (magenta) and cluster 2 (cyan), **F**: Alignment between AraC model and cluster 7 (red).

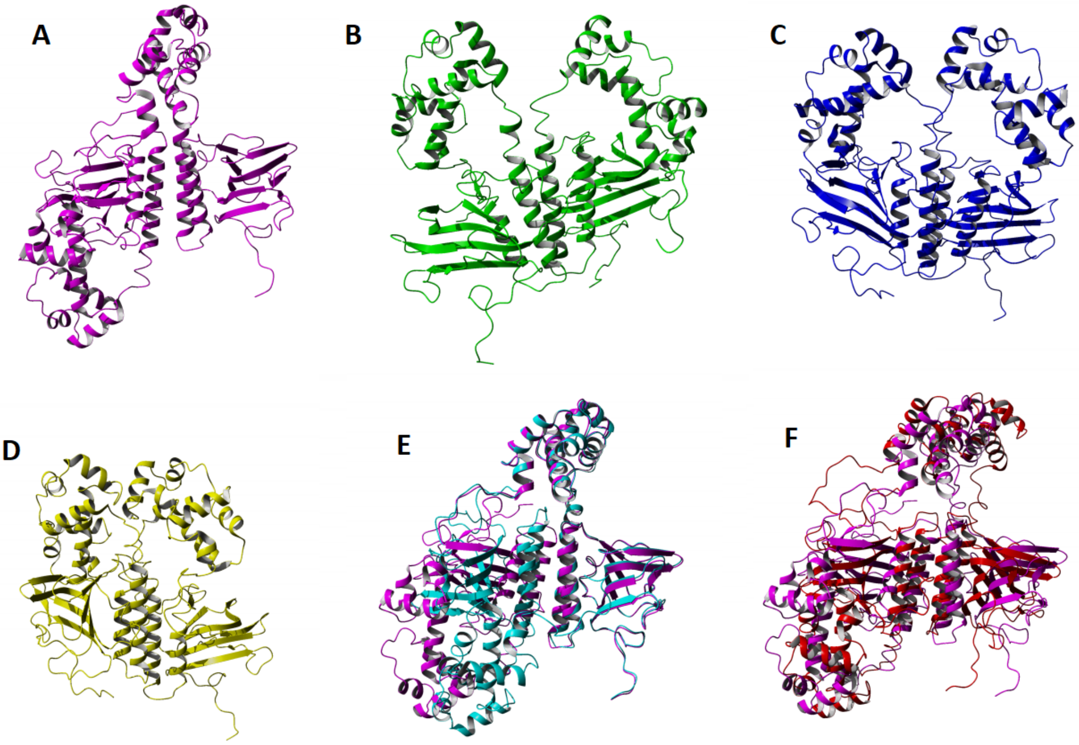

Fig. S31 AraC dimerization results of the HADDOCK webserver at pH 5. **A**: YASARA AraC homology hybrid model (magenta), **B**: cluster 5 (green), **C**: cluster 3 (blue), **D**: cluster 4 (yellow), **E**: Alignment between the AraC model (magenta) and cluster 1 (cyan), **F**: Alignment between the AraC model and cluster 2 (red).

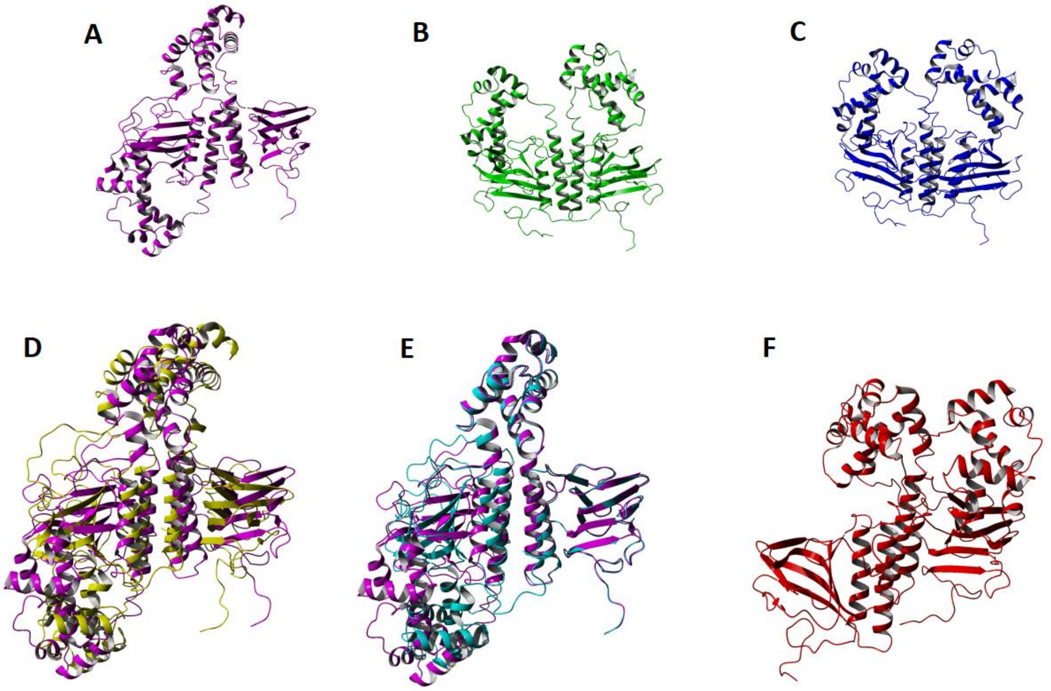

Fig. S32 AraC dimerization results of the HADDOCK webserver at pH 4. **A**: YASARA homology hybrid model (magenta), **B**: cluster 1 (green), **C**: cluster 3 (blue), **D**: Alignment between the AraC model (magenta) and cluster 2 (yellow), **E**: Alignment between the AraC model (magenta) and cluster 7 (cyan), **F**: cluster 8 (red).

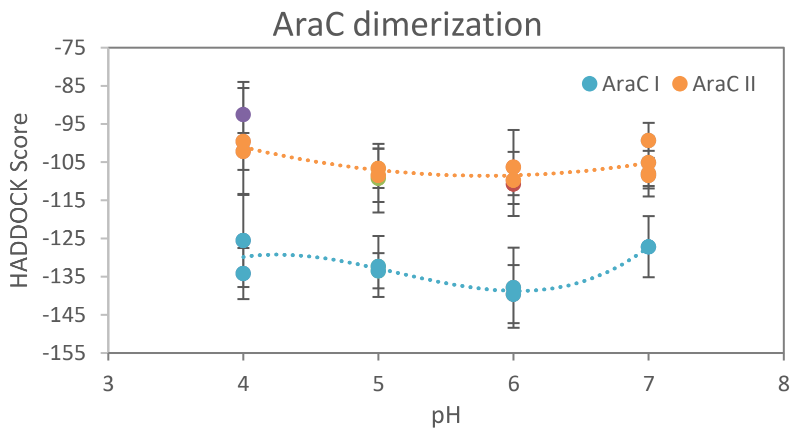

Fig. S33 AraC dimerization behaviour at different pH. Trend line AraC I is based on the clusters 6 (pH 7), 1 and 3 (pH 6), 5 and 3 (pH 5), and 1 and 3 (pH 4). A third-degree polynomial fitting was used. Trend line AraC II is based on clusters 2, 10, 1 and 4 (pH 7), 2 and 7 (pH 6), 1 and 2 (pH 5), and 2 and 7 (pH 4). A second-degree polynomial fitting was used.

Text S2.3 DNA binding and oligomerization behaviour of LacI at different pH values

For the docking simulation of LacI to its operator DNA, the YASARA hybrid dimer model was used. For pH 7, 6, 5, and 4 each, a unique cluster could be identified (Table S2). To validate the different docking poses, all structures were visually compared to the crystal structure with PDB ID 1EFA consisting of a LacI trimer bound to its operator sequence. For the alignment one monomer of the 1EFA structure was deleted.

Neither for pH 7, nor for pH 6, 5, or 4, a docking position modelled with the HADDOCK webserver correlated with the one determined by crystallization (Fig. S34-S37). Therefore, the different structures of each run were aligned and correlating docking poses were identified (Fig. S36). It was found, that the structures of the best scoring clusters for pH 7 (cluster 4), pH 6 (cluster 4), pH 5 (cluster 12) and pH 4 (cluster 9) were very similar (Fig. S38, A). A second set of well-aligning clusters were found, consisting of cluster 3 (pH 7), cluster 3 (pH 6), cluster 4 (pH 5) and cluster 5 (pH 4) (Fig. S38, B). These, however, exhibited lower scores that were within the standard deviation of other clusters. The two sets of clusters were used to predict binding behaviour of LacI at different pH values (Fig. S40).

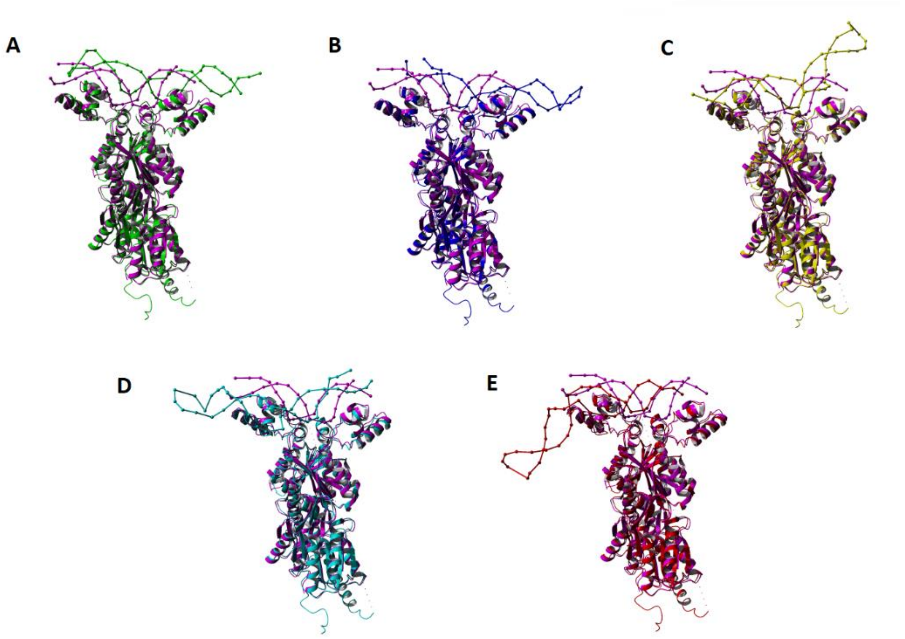

Fig. S34 LacI-DNA binding results of the HADDOCK webserver for pH 7. Alignment between the structure with PDB ID 1EFA (magenta) and the first structure of cluster 4 (**A**, green), cluster 3 (**B**, blue), cluster 8 (**C**, yellow), cluster 1 (**D**, cyan), and cluster 6 (**E**, red).

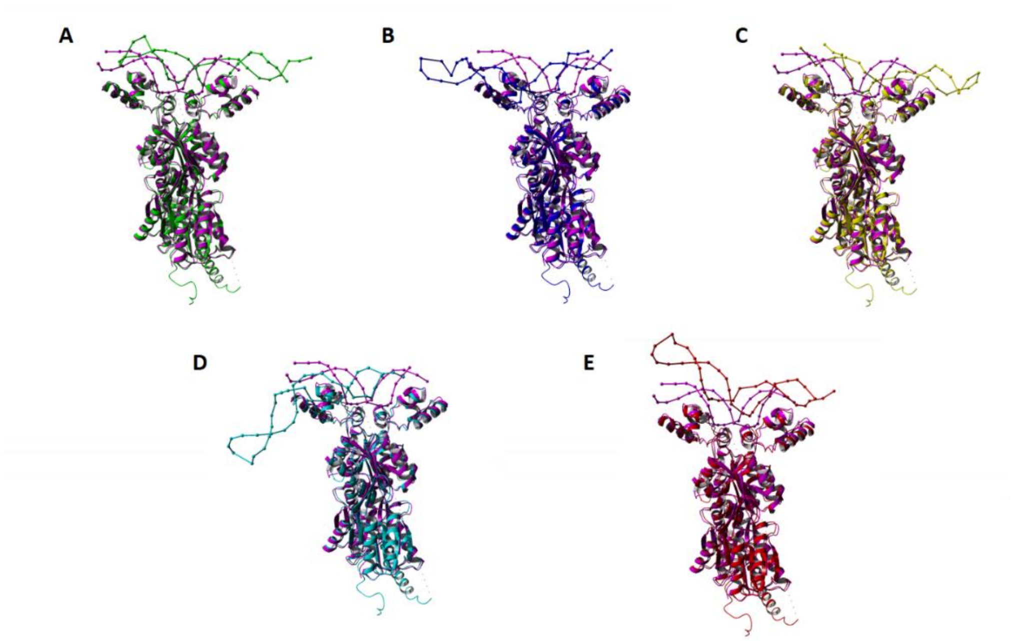

Fig. S35 LacI-DNA binding results of the HADDOCK webserver at pH 6. Alignment between the structure with PDB ID 1EFA (magenta) and the first structure of cluster 4 (**A**, green), cluster 1 (**B**, blue), cluster 3 (**C**, yellow), cluster 10 (**D**, cyan), and cluster 2 (**E**, red).

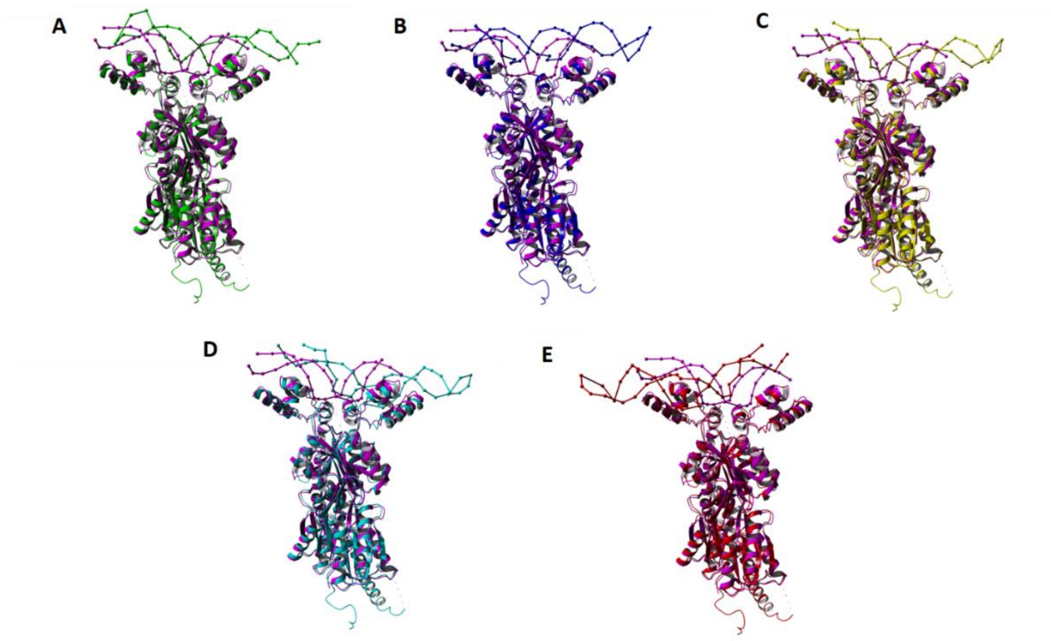

Fig. S36 LacI-DNA binding results of the HADDOCK webserver at pH 5. Alignment between the structure with PDB ID 1EFA (magenta) and the first structure of cluster 12 (**A**, green), cluster 1 (**B**, blue), cluster 11 (**C**, yellow), cluster 4 (**D**, cyan), and cluster 2 (**E**, red).

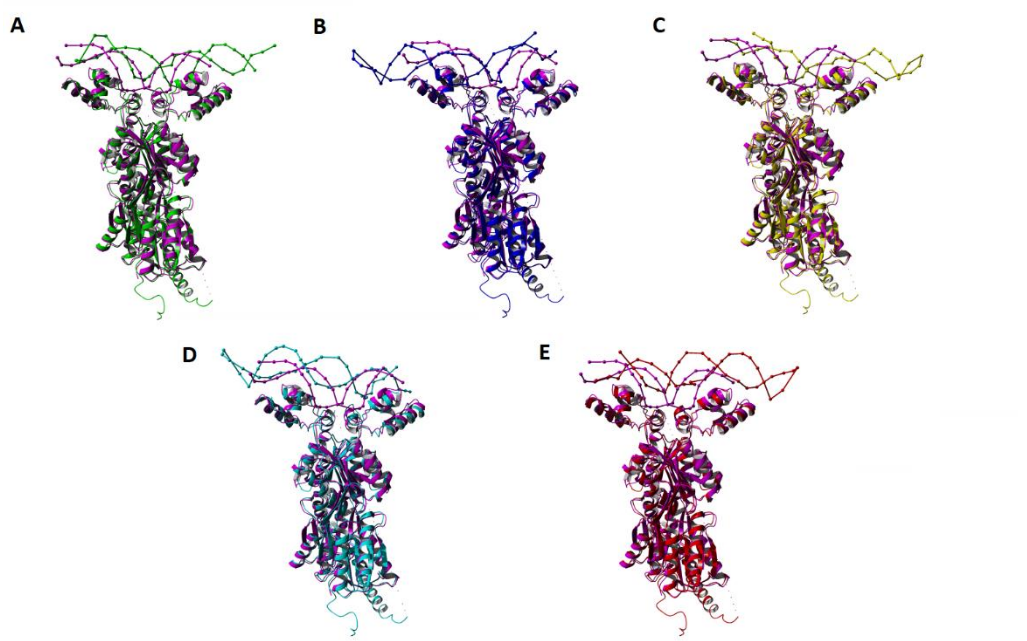

Fig. S37 LacI-DNA binding results of the HADDOCK webserver at pH 4. Alignment between the structure with PDB ID 1EFA (magenta) and the first structure of cluster 9 (**A**, green), cluster 1 (**B**, blue), cluster 5 (**C**, yellow), cluster 4 (**D**, cyan), and cluster 2 (**E**, red).

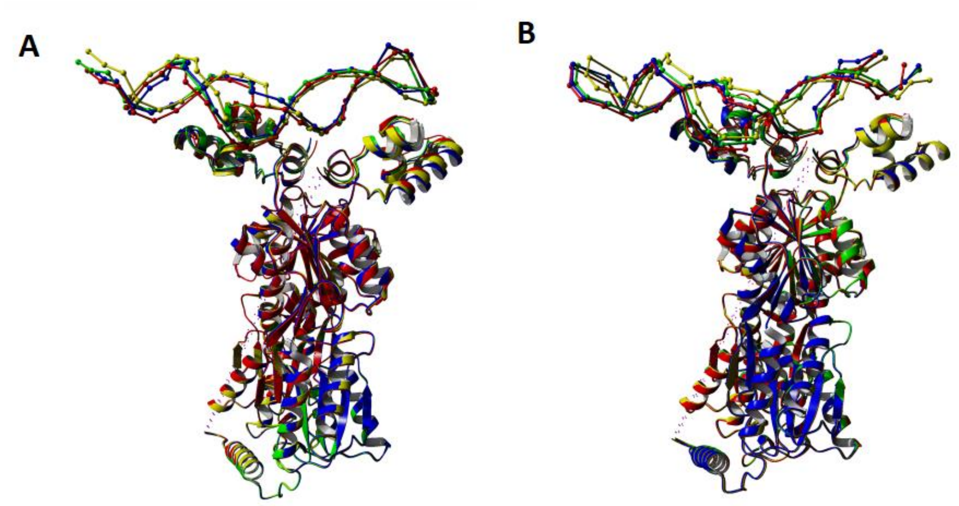

Fig. S38 Alignment of LacI docking poses. **A**: Alignment between cluster 4 (pH 7, red), cluster 4 (pH 6, green), cluster 12 (pH 5, blue), and cluster 9 (pH 4, yellow). **B**: Alignment between cluster 3 (pH 7, red), cluster 3 (pH 6, green), cluster 4 (pH 5, blue), and cluster 5 (pH 4, yellow).

For the first group of clusters (Fig. S39, LacI I) the HADDOCK scores increased with decreasing pH values, suggesting decreasing LacI-DNA binding. For the second group of clusters (Fig. S39, LacI II), however, the HADDOCK scores first increased and then decreased, following a parabolic trend. This suggested decreased LacI-DNA binding at pH 5 and 6, but unaltered LacI-DNA binding when comparing pH 7 and pH 4. Since no docking poses aligned with the crystal structure of PDB ID 1EFA it remained unclear, which pose and thereby which trend line is the most reliable one *in vivo*. However, the first set of clusters exhibited the most significant HADDOCK scores making a decreased LacI-DNA binding at decreased pH values a more trustworthy prediction.

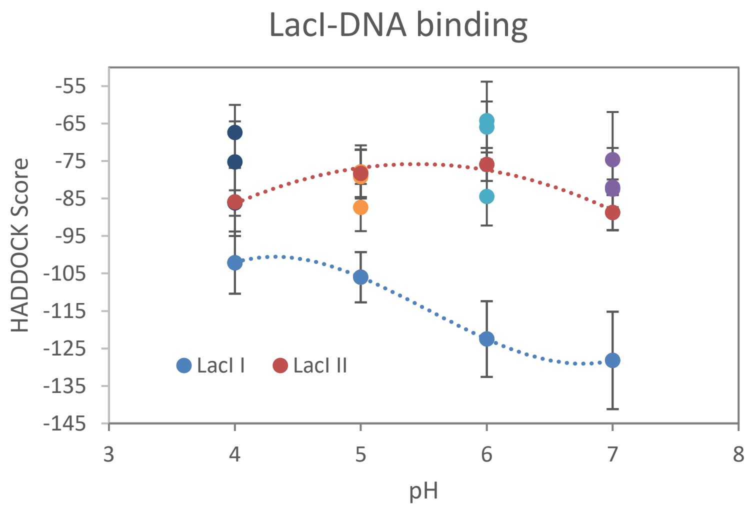

Fig. S39 LacI-DNA binding behaviour at different pH. Trend line LacI I is based on cluster 4 (pH 7) and cluster 4 (pH 6), cluster 12 (pH 5), and cluster 9 (pH 4). A third-degree polynomial fitting was used. Trend line LacI II is based on cluster 3 (pH 7), cluster 3 (pH 6), cluster 4 (pH 5), and cluster 5 (pH 4).A second-degree polynomial fitting was used.

For the tetramerization simulation, the dimers of the crystal structure with PDB ID 3EDC were used. The dimeric YASARA LacI hybrid model was missing crucial parts of the oligomerization region and was therefore not suitable for this simulation. For each pH value a unique cluster could be identified, i.e. cluster 2 for pH 7 and cluster 2 for pH 6, cluster 2 for pH 5, and cluster 2 for pH 4 (Table S2). These findings were supported by the visualized structures in which those clusters showed highest similarity with the crystal structure of the PDB ID 3EDC (Fig. S40-S43, A).

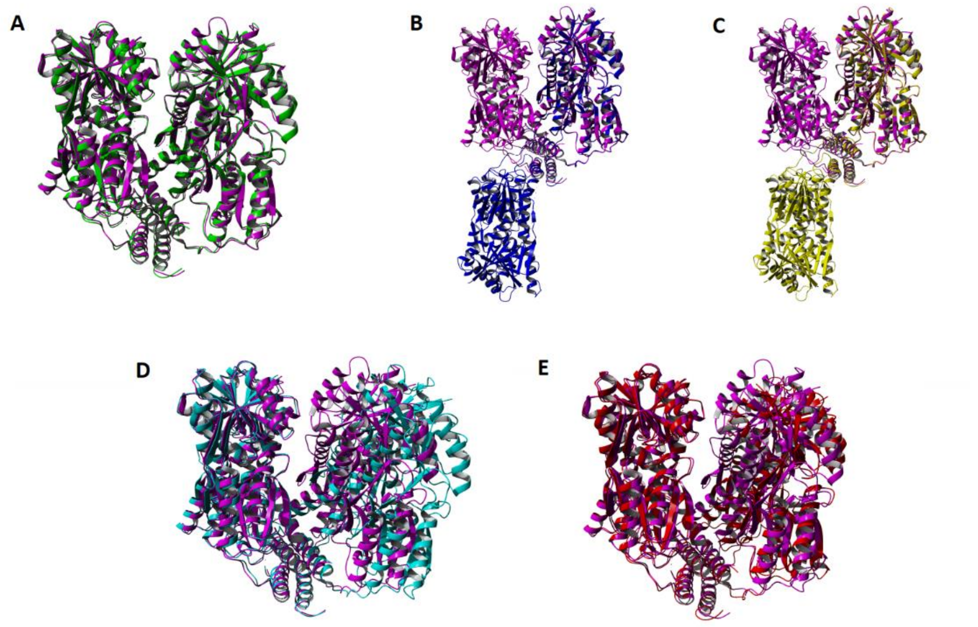

Fig. S40 LacI oligomerization results of the HADDOCK webserver at pH 7. Alignment between the structure with PDB ID 3EDC (magenta) and the first structure for cluster 2 (**A**, green), cluster 1 (**B**, blue), cluster 9 (**C**, yellow), cluster 5 (**D**, cyan), and cluster 4 (**E**, red).

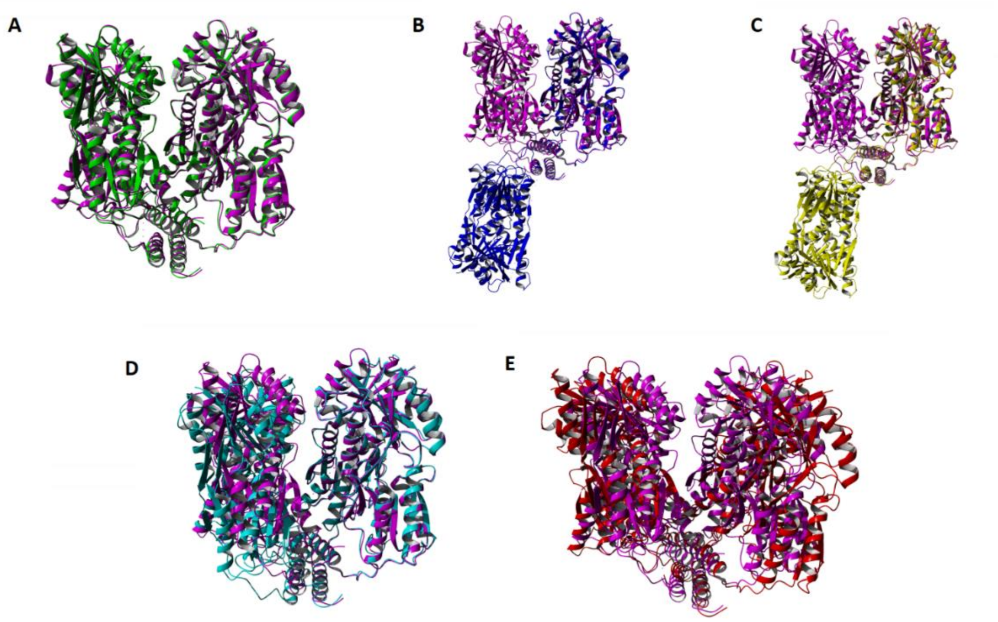

Fig. S41 LacI oligomerization results of the HADDOCK webserver at pH 6. Alignment between the structure with PDB ID 3EDC (magenta) with the first structure of cluster 2 (**A**, green), cluster 1 (**B**, blue), cluster 8 (**C**, yellow), cluster 4 (**D**, cyan), and cluster 5 (**E**, red).

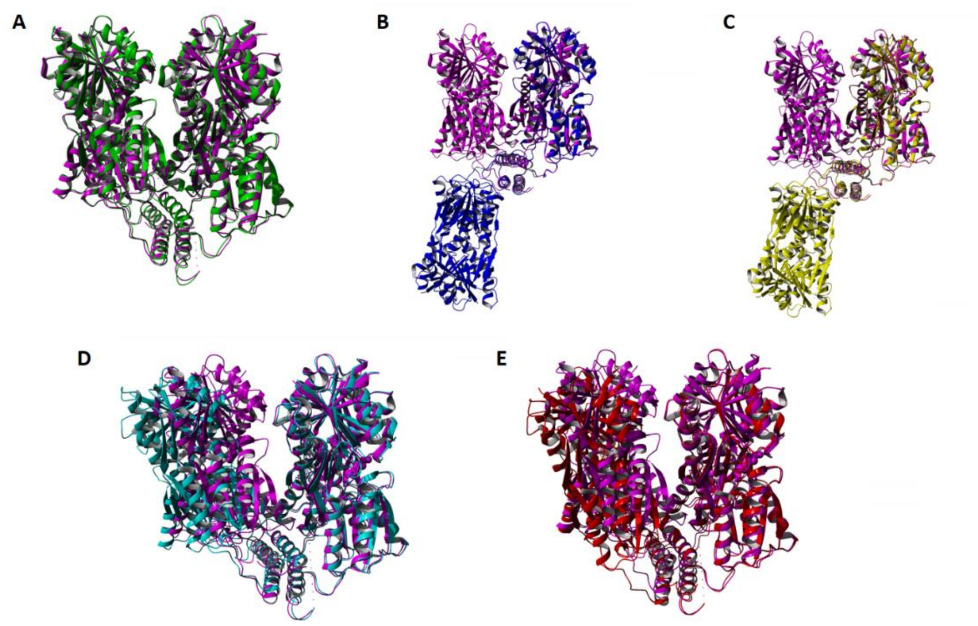

Fig. S42 LacI oligomerization results of the HADDOCK webserver at pH 5. Alignment between the structure with PDB ID 3EDC (magenta) and the first structure of cluster 2 (**A**, green), cluster 1 (**B**, blue), cluster 4 (**C**, yellow), cluster 8 (**D**, cyan), and cluster 3 (**E**, red).

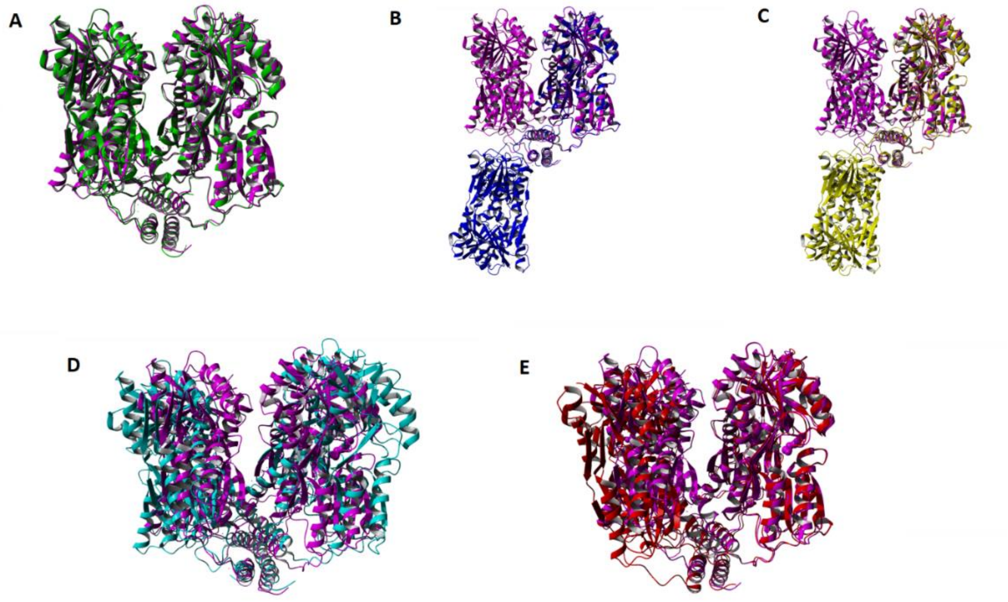

Fig. S43 LacI oligomerization results of the HADDOCK webserver at pH 4. Alignment between the structure with PDB ID 3EDC (magenta) and the first structure of cluster 2 (**A**, green), cluster 1 (**B**, blue), cluster 8 (**C**, yellow), cluster 3 (**D**, cyan), and cluster 5 (**E**, red).

The slightly increasing HADDOCK scores of this set of clusters suggested decreased binding of LacI dimers. However, it is arguable if the change has a significant effect on the tetramerization behaviour of LacI (Fig. S44).

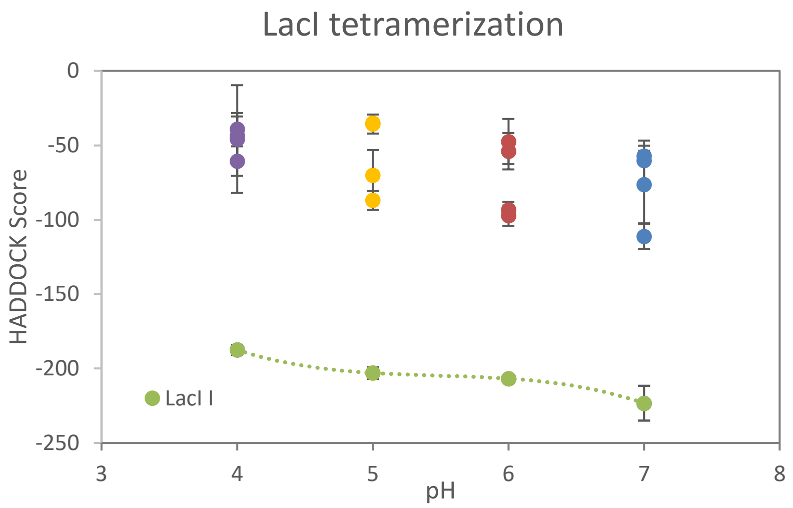

Fig. S44 LacI oligomerization behaviour at different pH. Trend line LacI I is based on cluster 2 (pH 7), cluster 2 (pH 6), cluster 2 (pH 5), and cluster 2 (pH 4). A third-degree polynomial fitting was used.

References

Krieger E, Koraimann G, Vriend G (2002) Increasing the precision of comparative models with YASARA NOVA - a self-parameterizing force field. Proteins 47(3):393-402 doi:10.1002/prot.10104

Sippl MJ (1993) Recognition of errors in 3-dimensional structures of proteins. Proteins 17(4):355-362 doi:10.1002/prot.340170404

van Zundert GCP, Rodrigues J, Trellet M, Schmitz C, Kastritis PL, Karaca E, Melquiond ASJ, van Dijk M, de Vries SJ, Bonvin A (2016) The HADDOCK2.2 web server: User-friendly integrative modeling of biomolecular complexes. J Mol Biol 428(4):720-725 doi:10.1016/j.jmb.2015.09.014
